# The first near-complete assembly of the hexaploid bread wheat genome, *Triticum aestivum*

**DOI:** 10.1093/gigascience/gix097

**Published:** 2017-10-23

**Authors:** Aleksey V Zimin, Daniela Puiu, Richard Hall, Sarah Kingan, Bernardo J Clavijo, Steven L Salzberg

**Affiliations:** Center for Computational Biology, McKusick-Nathans Institute of Genetic Medicine, Johns Hopkins University School of Medicine, Baltimore, MD 21205, USA; Institute for Physical Sciences and Technology, University of Maryland, College Park, MD 20742, USA; Pacific Biosciences, 1305 O'Brien Dr, Menlo Park, CA 94025, USA; Earlham Institute, Norwich Research Park Innovation Centre, Colney Ln, Norwich NR4 7UZ, UK; Departments of Biomedical Engineering, Computer Science, and Biostatistics, Johns Hopkins University, Baltimore, MD 21205, USA

**Keywords:** genome assembly, plant genomes, wheat genome, hybrid assembly, PacBio sequencing

## Abstract

Common bread wheat, *Triticum aestivum*, has one of the most complex genomes known to science, with 6 copies of each chromosome, enormous numbers of near-identical sequences scattered throughout, and an overall haploid size of more than 15 billion bases. Multiple past attempts to assemble the genome have produced assemblies that were well short of the estimated genome size. Here we report the first near-complete assembly of *T. aestivum*, using deep sequencing coverage from a combination of short Illumina reads and very long Pacific Biosciences reads. The final assembly contains 15 344 693 583 bases and has a weighted average (N50) contig size of 232 659 bases. This represents by far the most complete and contiguous assembly of the wheat genome to date, providing a strong foundation for future genetic studies of this important food crop. We also report how we used the recently published genome of *Aegilops tauschii*, the diploid ancestor of the wheat D genome, to identify 4 179 762 575 bp of *T. aestivum* that correspond to its D genome components.

## Introduction

For many years, the hexaploid (AABBDD) bread wheat genome, *Triticum aestivum*, has resisted efforts to sequence and assemble it. The first effort to sequence the genome, published in 2012 [1], used an earlier generation of sequencing technology and only assembled 5.42 billion bases (Gbp), approximately one-third of the genome. In a second attempt 2 years later, an international consortium published the results of a systematic effort to sequence the genome 1 chromosome at a time, using deep coverage in 100-bp Illumina reads [2]. That effort yielded a genome assembly containing only 10.2 billion bases of sequence, approximately two-thirds of the genome. The contiguity of this assembly was quite poor, with the 10.2 billion bases divided amongst hundreds of thousands of contigs, and with N50 sizes ranging from 1.7 to 8.9 kilobases (Kb) for the different chromosome arms. In 2017, a third assembly of wheat was published, estimated to represent 78% of the genome [3]. This assembly contained 12.7 billion bases of sequence, but it too was highly fragmented, containing more than 2.7 million contigs with an N50 contig size of 9731 bp and an N50 scaffold size of 64 267 bp.

The wheat genome's complexity, and the challenge it presents for genome assembly, stems not only from its large size (5 times the size of the human genome), but also from its very high proportion of relatively long, near-identical repeats, most of them due to transposable elements [4]. Because these repeats are much longer than the length of Illumina reads, efforts to assemble the genome using Illumina data have been unable to resolve these repeats. Another major challenge in assembling the wheat genome is that it is hexaploid, and the 3 component genomes–—wheat A, B, and D, each comprising 7 chromosomes—share many regions of high similarity. Genome assembly programs are thus faced with a doubly complex problem: first, that the genome is unusually repetitive and, second, that each chromosome exists in 6 copies with varying degrees of intra- and inter-chromosome similarity. All data for this assembly were generated from the Chinese spring variety (CS42, accession Dv418) of *T. aestivum*, which is highly inbred and thus nearly haploid, effectively reducing the number of copies of each chromosome from 6 to 3.

The most effective way to resolve repeats is to generate individual reads that contain them. If a single read is longer than a repeat, and if both ends of the read contain unique sequences, then genome assemblers can unambiguously place the repeat in the correct location. Without such reads, every long repeat creates a breakpoint in the assembly. Recent advances in sequencing, particularly the long read, single-molecule sequencing technologies from Pacific Biosciences (PacBio) and Oxford Nanopore (MinION), can produce reads in excess of 10 000 bp, although with a high error rate. By combining these very long reads with highly accurate shorter reads, we have been able to produce an assembly of the wheat genome with contigs that are more than 10 times longer than those produced in any previous attempt. Ours is the first assembly that contains nearly the entire length of the genome, with more than 15.3 billion bases.

Throughout this paper, we use 15.34 billion bases as the genome assembly size for computing the N50 statistics of different assemblies in order to make these statistics comparable. The true genome size of bread wheat has been estimated by flow cytometry to be close to 16 Gb [5]; based on this estimate, our assembly contains 96% of the genome sequence.

## Results

To create the wheat genome assembly, we generated 2 extremely large primary data sets. The first data set consisted of 7.06 billion Illumina reads containing approximately 1 trillion bases of DNA. The Illumina reads were 150-bp paired reads from short DNA fragments, averaging 400 bp in length. Using an estimated genome size of 15.3 Gbp, this represented 65-fold coverage of the genome. The second data set used Pacific Biosciences single-molecule (SMRT) technology to generate 55.5 million reads with an average read length just under 10 000 bp, containing a total of 545 billion bases of DNA, representing 36-fold coverage of the genome. All reads were generated from the Chinese spring variety (CS42, accession Dv418) of *T. aestivum*, the same variety as used in earlier attempts to sequence the genome.

### MaSuRCA assembly

To create the initial assembly, Triticum 1.0, we ran the MaSuRCA assembler (v. 3.2.1) on the full data set of Illumina and PacBio reads (MaSuRCA, RRID:SCR_010691). The first major step was the creation of super-reads [6] from the Illumina reads. Super-reads are highly accurate and longer than the original reads, and because they are fewer in number, they provide a means to greatly compress the original data. This step generated 95.7 million super-reads with a total length of 31 Gb, a mean size of 324 bp, and an N50 size of 474 bp (i.e., half of the total super-read sequence was contained in super-reads of 474 bp or longer). The super-reads provided a 32-fold compression of the original Illumina data.

Next we created *mega-reads* by using the super-reads to tile the PacBio reads, effectively replacing most PacBio reads (which have an average error rate of ∼15%) with much more accurate sequences [7]. Most PacBio reads were converted into a single mega-read, but in some cases a given PacBio read yielded 2 or more (shorter) mega-reads. In total, we created 57 020 767 mega-reads with a mean length of 4876 bp and an N50 length of 8427 bp. The total length of the mega-reads was 278 Gb, representing about ×18 genome coverage. As part of this step, we also created synthetic mate pairs; these link together 2 mega-reads when the pair of mega-reads originates from a single PacBio read. We generated these pairs by extracting 400 bp from opposite ends of each pair of consecutive mega-reads corresponding to a given PacBio read. This resulted in 23.45 million pairs of 400 bp reads, totalling 18.75 Gb.

Construction of super-reads and mega-reads required approximately 100 000 CPU hours, 95% of which was spent in the mega-reads step. By using large multi-core computers to run these steps in parallel, these steps took 1.5 months of elapsed (wall clock) time. The peak memory (RAM) usage was 1.2 terabytes.

We then assembled the mega-reads and the synthetic pairs using the Celera Assembler [8] (v. 8.3), which was modified to work with our parallel job scheduling system [9]. The CA assembly process required many iterations of the overlapping, error correction, and contig construction steps, and it was extremely time consuming, even with the many optimizations that have been incorporated into this assembler in recent releases. The total CPU time was ∼470 000 CPU hours (53.7 years), which was only made feasible by running it on a grid with thousands of jobs running in parallel (the maximum number was 3320) for some of the major steps. The total elapsed time was just over 5 months. When combined with the earlier steps, the entire assembly process took 6.5 months. The resulting assembly, labelled Triticum 1.0, contained 17.046 Gb in 829 839 contigs, with an N50 contig size of 76 267 bp and an N50 scaffold size of 101 195 bp (Table 1).

**Table 1:** Assembly statistics for each of the assemblies of *Triticum aestivum*, constructed as described in the text

Assembly	Element type	Number	Total size, bp	Average size, bp	N50 size, bp
Triticum 1.0	Contigs	829 839	17 045 571 778	20 541	76 267
	Scaffolds > 2 Kb	576 137	16 889 295 941	29 314	101 195
Triticum 2.0	Contigs	375 328	14 395 027 822	38 353	75 599
	Scaffolds > 2 Kb	252 501	14 412 484 332	57 078	100 805
FALCON Trit 1.0	Contigs	97 809	12 939 100 857	132 289	215 314
Triticum 3.0	Contigs	279 439	15 343 711 528	54 908	232 613
Triticum 3.1	Contigs	279 439	15 344 693 583	54 912	232 659

To enable fair comparisons, all N50 sizes were computed using an estimated genome size of 15.34 Gb. Next, in order to detect and remove redundant regions of the assembly, we aligned the assembly against itself using the nucmer program from the MUMmer package [13]. We identified and excluded scaffolds that were completely contained in and ≥96% identical to other scaffolds. After this de-duplication procedure, the reduced assembly, Triticum 2.0, contained 14.40 Gbp in 375 328 contigs with an N50 contig size of 75 599 bp, with scaffolds spanning 14.45 Gbp and an N50 scaffold size of 100 805 bp (Table 1).

### FALCON assembly

Independently of the MaSuRCA assembly, we assembled the PacBio data alone using the FALCON assembler [10], followed by polishing with the Arrow program, which substantially improves the consensus accuracy. FALCON implements a hierarchical assembly approach; the initial step is to error-correct long reads by aligning all reads to a subset of the longest reads. Given the relatively low raw read coverage (×36), we used a long-read cutoff of 1 Kb, generating ×11 coverage of error-corrected reads with an N50 size of 16 Kb. Error correction and assembly of the corrected reads were completed using ∼150 000 CPU hours, which took ∼3 weeks on a 16-node cluster. The contigs output from FALCON required further polishing, which involves realignment of raw reads and calculation of a new consensus [11]. For the polishing step, we used Pacbio's resequencing pipeline from the SMRT Analysis package [12] after first splitting the assembled contigs into <4-Gbp chunks (a limit of the aligner). Polishing required an additional ∼160 000 CPU hours, for a total of 310 000 CPU hours and 6 weeks’ elapsed (wall clock) time.

These steps produced an assembly, designated FALCON Trit 1.0, containing 12.94 Gbp in 97 809 contigs with a mean size of 132 289 and an N50 size of 215 314 bp (Table 1).

### Merged assembly

**Figure 1:**
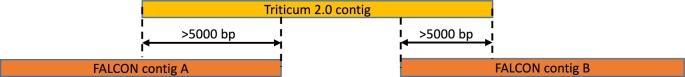
Illustration of the merging process for the Triticum 2.0 and FALCON Trit 1.0 assemblies. If 2 contigs A and B from the FALCON assembly overlapped a Triticum 2.0 contig by at least 5000 bp, then A and B were merged together using the Triticum 2.0 contig to fill the gap.

The contigs from the FALCON assembly were larger than those from the MaSuRCA assembly; however, the total size of the assembly was 1.5 Gbp smaller. To capture the advantages of both assemblies, we merged them as follows. We aligned the contigs (not scaffolds) from the 2 assemblies using MUMmer 4.0 [13] and extracted all pairwise best matches. We then merged each pair of FALCON contigs when they overlapped a single Triticum 2.0 contig by at least 5000 bp, with Triticum 2.0 sequence filling the gap (see Fig. 1).

After merging and extending the FALCON contigs, we then identified all MaSuRCA scaffolds that were not contained in the longer FALCON contigs and added these to the new assembly. The resulting merged assembly, Triticum 3.0, contains 15 343 750 409 bp in 279 529 contigs, with a contig N50 size of 232 613 bp (Table 1). The longest contig is 4 510 883 bp. The assembly contains no unknown (N) bases.

### Genome complexity

As described above, previous attempts to assemble the hexaploid wheat genome were stymied because of its exceptionally high repetitiveness, but until now we had no reliable way to quantify how repetitive the genome truly is. To answer this question with a precise metric, we computed the k-mer uniqueness ratio, a metric defined earlier as a way to capture repetitiveness that reflects the difficulty of assembly [14]. This ratio is defined as the percentage of a genome that is covered by unique sequences of length *k* or longer. If, for example, 90% of a genome is comprised of unique 50-mers, then one might expect that 90% of that genome could be assembled using accurate (low–error rate) reads that were longer than 50 bp.

**Figure 2:**
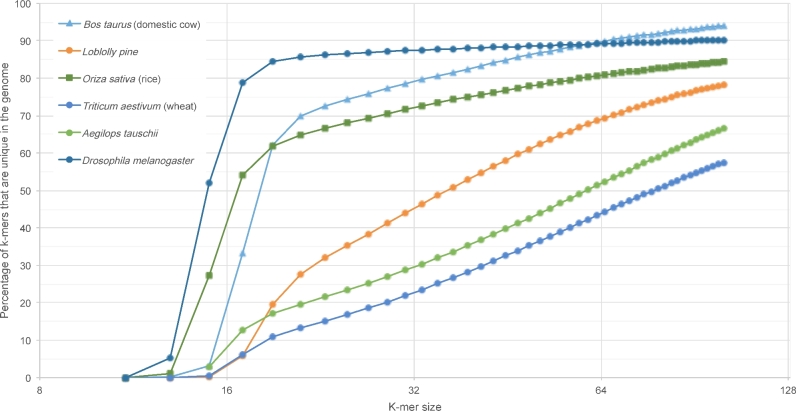
K-mer uniqueness ratios for the wheat genome (*Triticum aestivum*) compared to the cow, fruit fly, rice, loblolly pine, and *Ae. tauschii* genomes. The plot shows the percentage of each genome that is covered (y-axis) by unique sequences of length k for various values of k (x-axis).

With the Triticum 3.0 assembly in hand, we computed the k-mer uniqueness ratio for wheat and compared it to several other plant and animal genomes, as shown in Fig. 2. As the figure illustrates, for any value of k, a much smaller percentage of the wheat genome is covered by unique k-mers than other plant or animal genomes, with the exception of *Ae. tauschii*, which, as expected (because it is nearly identical to the D genome of hexaploid *T. aestivum*), is only slightly less repetitive. For example, only 44% of the 64-mers in the wheat genome are unique, as contrasted with 90% of the 64-mers in cow and 81% of the 64-mers in rice. This analysis demonstrates that in order to obtain an assembly covering most of the wheat genome, particularly if the algorithm relies on de Bruijn graphs, much longer reads will be required. Our sequencing strategy, by using deep coverage in very long PacBio reads coupled with highly accurate Illumina reads, was able to produce the long, accurate reads required to assemble this very complex genome.

### Identifying the wheat D genome

*T. aestivum* is a hexaploid plant with 3 diploid ancestors, one of which is *Aegilops tauschii*, commonly known as goat grass. *Ae. tauschii* itself is a highly repetitive genome that has resisted attempts at assembly, but we recently published a highly contiguous draft assembly (Aet_MR 1.0) using a similar strategy to the one used for wheat, a combination of PacBio and Illumina sequences [7]. *T. aestivum*'s hexaploid composition is typically represented as AABBDD, where the D genome was contributed by an ancestor of *Ae. tauschii*. The hexaploidization event occurred very recently, approximately 8000 years ago, when *Ae. tauschii* spontaneously hybridized with a tetraploid wheat species, *Triticum turgidum* [15].

Because this event was so recent, the wheat D genome and *Ae. tauschii* are highly similar, much closer to one another than the D genome is to either the A or B genomes. We used this similarity to identify the D genome components of our assembly by aligning the *Ae. tauschii* contigs in Aet_MR 1.0 to Triticum 3.0. We used the nucmer program [13] to identify all alignments representing best matches between Triticum 3.0 and Aet_MR 1.0 with a minimum identity of 97%. The vast majority of the 2 genomes are >99% identical, making this filtering process relatively straightforward.

After filtering, we identified 50 101 contigs with a total length of 4 179 762 575 bp from Triticum 3.0 that aligned to *Ae. tauschii*. We separated these D genome contigs from Triticum 3.0 and provided them as the first release of the wheat D genome, which we have named TriticumD 1.0. The N50 size of these contigs is 224 953 bp, using a genome size estimate of 4.18 Gb for wheat D. The total size of 4.18 Gb corresponds closely to the 4.33 Gb in the recently published *Ae. tauschii* (Aet_MR 1.0) assembly [7].

We also ran the alignments in the other direction, aligning all of Aet_MR 1.0 to TriticumD 1.0, and found that 99.8% of the *Ae. tauschii* assembly matches TriticumD; only 8.96 Mb failed to align. The overall mapping is complex; although most of the *Ae. tauschii* and wheat D genomes align in a 1-to-1 mapping, many scaffolds align in a many-to-one or one-to-many arrangement. Thus the additional 150 Mb in *Ae. tauschii* appears to be due to gain/loss of repeats rather than loss of unique sequence from wheat D.

### Assembly quality and completeness

Assessing the quality of an assembly is challenging, especially when the previous assemblies are so much more fragmented, as they are in the case of *T. aestivum*. However, the very high-fidelity alignments between Triticum 3.0 and the published *Ae. tauschii* genome, at over 99% identity, provide strong support for Triticum 3.0’s accuracy. We found no large-scale structural disagreements between the assemblies, other than the many-to-one mappings for some of the scaffolds. These could indicate that one assembly has over-collapsed a repeat, but they could also indicate a true polymorphism; we do not have sufficient data to distinguish these possibilities. The fact that 99.8% of *Ae. tauschii* aligns to Triticum 3.0 supports the hypothesis that the assembly is largely complete as well.

As a further evaluation of assembly quality, we aligned 19 401 BAC ends from the wheat chromosome 3B-specific BAC library, TA3B (NCBI BioSample SAMN001187987) [16], to all contigs in Triticum 3.1. A total of 18 465 BAC ends aligned, of which 2739 pairs aligned to the same contig. Of these 2739 pairs, 2709 (99%) aligned in the correct orientation with a distance consistent with the mean size for the library. In no case did a pair of BAC ends align to a single contig in the wrong orientation. Out of all BACs where the ends aligned to different contigs, only 282 had 1 BAC end aligning sufficiently far from a contig's end to permit the other BAC end to align to the same contig; these could represent mis-assembled contigs, but they could also be explained by unusually long BACs or alignment artifacts.

We used BUSCO (version 3.0.2; BUSCO, RRID:SCR_015008) [17] to assess the completeness of the Triticum 3.1 assembly based on the presence of the single-copy orthologs from the OrthoDB (v. 9.1) database (OrthoDB, RRID:SCR_011980) [18]. We found that 1415 out of 1440 BUSCO genes are present and complete in the Triticum 3.1 assembly, of which 161 are single-copy and 1254 are in multiple copies. The large number of duplicated genes is likely due to the polyploidy of the genome. Only 4 BUSCO genes are fragmented and 21 are missing. We ran the same analysis on the most complete published bread wheat assembly, TGACv1 [3], and found that it contains 1411 complete BUSCO genes (very slightly fewer than Triticum 3.1), of which 126 are single-copy, 1285 are multiple-copy, 8 are fragmented, and 21 are missing.

### Re-polishing to create Triticum 3.1

Finally, we used an independent set of Illumina 250-bp reads from an earlier study [3] to measure the quality of the consensus sequence. We used the KAT program [19] to count all 31-mers in each assembly and compare these counts with the 31-mers in the read data. Because the read data here represented 30-fold coverage of the genome, 31-mers that occur approximately 30 times should represent unique sequences; i.e., they are expected to occur exactly once in the assembly.

**Figure 3:**
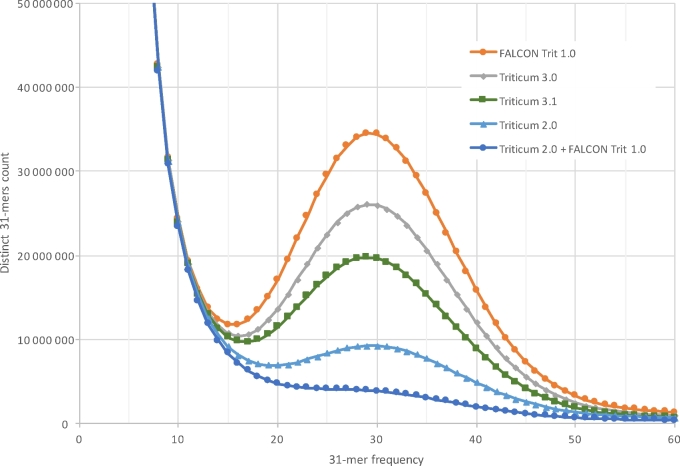
Missing 31-mers in the different assemblies of *Triticum aestivum*. Using the Illumina read data from a previously published assembly of the same genome, we counted all 31-mers in the reads and then plotted how many of these 31-mers are missing from each assembly. The x-axis shows how often the k-mers occur in the reads. The y-axis shows how many distinct k-mers are missing from each assembly. The FALCON Trit 1.0 assembly had the most missing k-mers, while the MaSuRCA-driven Triticum 2.0 assembly had the fewest.

The KAT analysis revealed that the FALCON Trit 1.0 assembly was missing a relatively large number of 31-mers that occurred in the reads (Fig. 3), while the MaSuRCA-derived Triticum 2.0 assembly was missing far fewer of these 31-mers. The Triticum 3.0 assembly, which used the polished FALCON contigs for most of its consensus sequence, was also missing many 31-mers. The most likely explanation for this effect is that the polishing process over-corrected by replacing some 31-mers with near-identical ones. This would have the effect of creating an excess of 31-mers that occur exactly twice in the assembly, although their coverage indicated that they should occur once. The KAT analysis confirmed this expectation.

Because Triticum 2.0 had far fewer missing 31-mers, and because it created its consensus from mega-reads whose sequence was based on Illumina data, we re-polished Triticum 3.0 by aligning it to Triticum 2.0, extracting the mutual best matches, and then using the 2.0 sequence as the final consensus. This allowed us to replace about 98% of the Falcon consensus in the 3.0 assembly with the higher-quality MaSuRCA consensus. The resulting assembly, Triticum 3.1, has exactly the same number of contigs and scaffolds (Table 1) but has an improved overall consensus, containing more of the true 31-mers (Fig. 3). Because of changes in the consensus sequence, the 3.1 assembly is very slightly larger as well. To evaluate the possibility of further improvements, we analysed the 31-mer spectra of both FALCON Trit 1.0 and Triticum 2.0 as a single sequence set. We found that this almost completely eliminated the missing 31-mers (Fig. 3), illustrating that further improvements in the consensus are possible and are planned for future assembly releases.

## Discussion

In 2004, an international consortium determined that whole-genome shotgun (WGS) sequencing of hexaploid wheat was simply too difficult, "mainly because of the large size and highly repetitive nature of the wheat genome" [20]. The consortium instead determined that the chromosome-by-chromosome approach would be more effective. This strategy, which was far slower and more costly than WGS sequencing, produced a genome assembly that was highly fragmented and that contained only 10.2 Gb [2].

The assembly described here is the first to successfully reconstruct essentially all of the hexaploid wheat genome, *Triticum aestivum*, and to produce relatively large contiguous sequences. The final assembly contains 15 344 693 583 bp with an N50 contig size of 232 659 bp. The previous chromosome-based assembly was not only much smaller overall, but it had average contig sizes approximately 50 times smaller [2]. A recent whole-genome assembly based on deep Illumina sequencing contained 2 726 911 contigs spanning 12 658 314 504 bp and had a contig N50 size of 9731 bp [3]. Compared to Triticum 3.0, that assembly is 2.69 Gb smaller, and its contigs are 24 times smaller. (Note that in order to provide a fair comparison, all N50 sizes reported here are based on the same 15.34-Gb total genome size.)

Why did previous attempts to assemble *T. aestivum* produce a result that was billions of nucleotides shorter than the true genome size? The most likely explanation is that the repetitive sequences, which cover some 90% of the genome [4, 20], are so similar to one another that genome assembly programs cannot avoid collapsing them together. This is a well-known problem for genome assembly, particularly when using the short reads produced by next-generation sequencing technologies. If the differences between repeats occur at a lower rate than sequencing errors, then assemblers cannot distinguish them. The result is an assembly that is both highly fragmented and too short. The same phenomenon can be seen in attempts to assemble *Ae. tauschii* from short reads. An assembly of that genome using Illumina and 454 sequencing data contained only 2.69 Gb and had an N50 contig size of just 2.1 Kb [15]. A hybrid assembly using both Illumina and PacBio data, reported by our group early in 2017, produced an assembly of 4.33 Gb, closely matching the estimated genome size, with a contig N50 size of 487 Kb [7].

The key factor in producing a true draft assembly for this exceptionally repetitive genome was the use of very long reads, averaging just under 10 000 bp each, which were required to span the long, ubiquitous repeats in the wheat genome. Deep coverage in these reads (×36, or 545 Gb of raw sequence), coupled with even deeper coverage (×65) in low–error rate short reads, allowed us to produce a highly accurate and highly contiguous consensus assembly. The massive data set, more than 1.5 trillion bases, also required an unprecedented amount of computing power to assemble, and its completion would not have been possible without the availability of very large parallel computing grids. All together, the various assembly steps took 880 000 CPU hours, or just over 100 CPU years. An important technical note is that the computational cost was not simply a function of genome size, but more critically a function of its repetitiveness. The presence of large numbers of unusually long exact and near-exact repeats (Fig. 2) means that all of these sequences overlap one another, leading to a quadratic increase in the number of sequence alignments that an assembler must consider.

Finally, ours is the first assembly to cleanly separate the D genome component from the A and B genomes of hexaploid wheat by aligning this assembly to the draft genome of *Aegilops tauschii*, the progenitor of the wheat D genome. This separation was possible because *Ae. tauschii* is much closer to wheat D, having diverged approximately 8000 years ago [20], than either genome is to wheat A or B.

The wheat genome presented here provides, for the first time, a near-complete substrate for future studies of this important food crop. Previous efforts to annotate the genome have been hampered by the absence of a large proportion of the genome itself, making inferences about missing genes or gene families difficult, and also by the highly fragmented nature of previous assemblies, which had average contig sizes under 10 Kb. With more than half of the genome now contained in contigs longer than 232 Kb, the Triticum 3.0 assembly will contain many more genes within single contigs, greatly aiding future efforts, which are already under way, to study its gene content, evolution, and relationship to other plant species.

## Availability of data

The Triticum project data have been deposited at the National Center for Biotechnology Information (NCBI) under BioProject PRJNA392179. The assembly has been deposited at DDBJ/ENA/GenBank under accession number NMPL00000000. The version described in this paper is version NMPL01000000. The PacBio and Illumina reads are available under the same BioProject. The TriticumD 1.0 contigs are available separately at ftp://ftp.ccb.jhu.edu/pub/data/Triticum_aestivum/Wheat_D_genome. The preliminary assemblies described in the paper and other supporting files are also available in the *GigaScience* database, *Giga*DB [21].

## Competing interests statement

Two of the authors are employees of Pacific Biosciences. None of the other authors has any competing or conflicting interests.

## Supplementary Material

GIGA-D-17-00164_Original-Submission.pdf

GIGA-D-17-00164_Revision-1.pdf

GIGA-D-17-00164_Revision-2.pdf

Response-to-Reviewer-Comments_Original-Submission.pdf

Response-to-Reviewer-Comments_Revision-1.pdf

Response-to-reviewers-82517.pdf

Reviewer-1-Report-(Original-Submission).pdf

Reviewer-2-Report-(Original-Submission).pdf

Reviewer-3-Report-(Original-Submission).pdf

Reviewer-4-Report-(Revision-1).pdf

## Abbreviations

bp: base pair; Gb: gigabases; Kb: kilobases; WGS: whole-genome sequencing; BAC: bacterial artificial chromosome; CPU: central processing unit.
