# Supplementary material for: The first near-complete assembly of the hexaploid bread wheat genome, Triticum aestivum
Source: Gigascience. 2017 Oct 23;6(11):1–7. doi: 10.1093/gigascience/gix097 (PMC5691383; doi:10.1093/gigascience/gix097)
Supplement: GIGA-D-17-00164_Revision-2.pdf [file gix097_giga-d-17-00164_revision-2.pdf]

GigaScience

The first near-complete assembly of the hexaploid bread wheat genome, Triticum aestivum

--Manuscript Draft--

**Manuscript Number:**	GIGA-D-17-00164R2
**Full Title:**	The first near-complete assembly of the hexaploid bread wheat genome, Triticum aestivum
**Article Type:**	Data Note
**Funding Information:**	Directorate for Biological Sciences (IOS-1238231) — Not applicable<br>National Human Genome Research Institute (R01HG006677) — Dr. Steven L. Salzberg<br>Directorate for Biological Sciences (IOS-1444893) — Dr. Aleksey V. Zimin
**Abstract:**	Common bread wheat, Triticum aestivum, has one of the most complex genomes known to science, with 6 copies of each chromosome, enormous numbers of near-identical sequences scattered throughout, and an overall haploid size of more than 15 billion bases. Multiple past attempts to assemble the genome have produced assemblies that were well short of the estimated genome size. Here we report the first near-complete assembly of T. aestivum, using deep sequencing coverage from a combination of short Illumina reads and very long Pacific Biosciences reads. The final assembly contains 15,344,693,583 bases and has a weighted average (N50) contig size of 232,659 bases. This represents by far the most complete and contiguous assembly of the wheat genome to date, providing a strong foundation for future genetic studies of this important food crop. We also report how we used the recently published genome of Aegilops tauschii, the diploid ancestor of the wheat D genome, to identify 4,179,762,575 bp of T. aestivum that correspond to its D genome components.
**Corresponding Author:**	Steven L. Salzberg, Ph.D.<br>Johns Hopkins University<br>Baltimore, MD UNITED STATES
**Corresponding Author Secondary Information:**	
**Corresponding Author's Institution:**	Johns Hopkins University
**Corresponding Author's Secondary Institution:**	
**First Author:**	Aleksey V. Zimin, Ph.D.
**First Author Secondary Information:**	
**Order of Authors:**	Aleksey V. Zimin, Ph.D.<br>Daniela Puiu, M.S.<br>Richard Hall, Ph.D.<br>Sarah Kingan, Ph.D.<br>Bernardo J. Clavijo<br>Steven L. Salzberg, Ph.D.
**Order of Authors Secondary Information:**	
**Response to Reviewers:**	This is the final version with a few extra edits included by the Editor.
**Additional Information:**	
**Question**	**Response**

Powered by Editorial Manager® and ProduXion Manager® from Aries Systems Corporation

Are you submitting this manuscript to a special series or article collection?	No
**Experimental design and statistics**<br><br>Full details of the experimental design and statistical methods used should be given in the Methods section, as detailed in our Minimum Standards Reporting Checklist. Information essential to interpreting the data presented should be made available in the figure legends.<br><br>Have you included all the information requested in your manuscript?	Yes
**Resources**<br><br>A description of all resources used, including antibodies, cell lines, animals and software tools, with enough information to allow them to be uniquely identified, should be included in the Methods section. Authors are strongly encouraged to cite Research Resource Identifiers (RRIDs) for antibodies, model organisms and tools, where possible.<br><br>Have you included the information requested as detailed in our Minimum Standards Reporting Checklist?	Yes
**Availability of data and materials**<br><br>All datasets and code on which the conclusions of the paper rely must be either included in your submission or deposited in publicly available repositories (where available and ethically appropriate), referencing such data using a unique identifier in the references and in the "Availability of Data and Materials" section of your manuscript.<br><br>Have you have met the above requirement as detailed in our Minimum Standards Reporting Checklist?	Yes

Manuscript
Click here to download Manuscript wheat-genome-assembly_Gigascience_revised_082517(SCE).docx

The first near-complete assembly of the hexaploid bread wheat genome, *Triticum aestivum*

Aleksey V. Zimin[1,2], Daniela Puiu[1], Richard Hall[3], Sarah Kingan[3], Bernardo J. Clavijo[4], and Steven L. Salzberg[1,5,*]

[1]Center for Computational Biology, McKusick-Nathans Institute of Genetic Medicine, Johns Hopkins University School of Medicine, Baltimore, MD
[2]Institute for Physical Sciences and Technology, University of Maryland, College Park, MD
[3]Pacific Biosciences, Menlo Park, CA
[4]Earlham Institute, Norwich NR4 7UZ, United Kingdom
[5]Departments of Biomedical Engineering, Computer Science, and Biostatistics, Johns Hopkins University, Baltimore, MD
[*]To whom correspondence should be addressed: salzberg@jhu.edu.

ORCID IDs:
Aleksey V. Zimin: 0000-0001-5091-3092
Bernardo J. Clavijo: 0000-0002-7597-2774
Steven L. Salzberg: 0000-0002-8859-7432

Abstract

Common bread wheat, *Triticum aestivum*, has one of the most complex genomes known to science, with 6 copies of each chromosome, enormous numbers of near-identical sequences scattered throughout, and an overall haploid size of more than 15 billion bases. Multiple past attempts to assemble the genome have produced assemblies that were well short of the estimated genome size. Here we report the first near-complete assembly of *T. aestivum*, using deep sequencing coverage from a combination of short Illumina reads and very long Pacific Biosciences reads. The final assembly contains 15,344,693,583 bases and has a weighted average (N50) contig size of 232,659 bases. This represents by far the most complete and contiguous assembly of the wheat genome to date, providing a strong foundation for future genetic studies of this important food crop. We also report how we used the recently published genome of *Aegilops tauschii*, the diploid ancestor of the wheat D genome, to identify 4,179,762,575 bp of *T. aestivum* that correspond to its D genome components.

Introduction

For many years, the hexaploid (AABBDD) bread wheat genome, *Triticum aestivum,* has resisted efforts to sequence and assemble it. The first effort to sequence the genome, published in 2012 [1], used an earlier generation of sequencing technology and only assembled 5.42 billion bases (Gbp), approximately one-third of the genome. In a second attempt two years later, an international consortium published the results of a systematic effort to sequence the genome one chromosome at a time, using deep coverage in 100-bp Illumina reads [2]. That effort yielded a genome assembly containing only 10.2 billion bases of sequence, approximately two-thirds of the genome. The contiguity of this assembly was quite poor, with the 10.2 billion bases divided amongst hundreds of thousands of contigs, and with N50 sizes ranging from 1.7 to 8.9 kilobases (Kb) for the different chromosome arms. In 2017, a third assembly of wheat was published, estimated to represent 78% of the genome [3]. This assembly contained 12.7 billion bases of sequence, but it too was highly fragmented, containing over 2.7 million contigs with an N50 contig size of 9,731 bp and an N50 scaffold size of 64,267 bp.

The wheat genome's complexity, and the challenge it presents for genome assembly, stems not only from its large size (five times the size of the human genome), but also from its very high proportion of relatively long, near-identical repeats, most of them due to transposable elements [4]. Because these repeats are much longer than the length of Illumina reads, efforts to assemble the genome using Illumina data have been unable to resolve these repeats. Another major challenge in assembling the wheat genome is that it is hexaploid, and the three component genomes–wheat A, B, and D, each comprising seven chromosomes–share many regions of high similarity. Genome assembly programs are thus faced with a doubly complex problem: first that

the genome is unusually repetitive, and second that each chromosome exists in six copies with varying degrees of intra- and inter-chromosome similarity. All data for this assembly was generated from the Chinese spring variety (CS42, accession Dv418) of *T. aestivum*, which is highly inbred and thus nearly haploid, effectively reducing the number of copies of each chromosome from six to three.

The most effective way to resolve repeats is to generate individual reads that contain them. If a single read is longer than a repeat, and if both ends of the read contain unique sequences, then genome assemblers can unambiguously place the repeat in the correct location. Without such reads, every long repeat creates a breakpoint in the assembly. Recent advances in sequencing, particularly the long read, single-molecule sequencing technologies from Pacific Biosciences (PacBio) and Oxford Nanopore (MinION), can produce reads in excess of 10,000 bp, although with a high error rate. By combining these very long reads with highly accurate shorter reads, we have been able to produce an assembly of the wheat genome with contigs that are more than ten times longer than those produced in any previous attempt. Ours is the first assembly that contains nearly the entire length of the genome, with more than 15.3 billion bases.

Throughout this paper we use 15.34 billion bases as the genome assembly size for computing the N50 statistics of different assemblies, in order to make these statistics comparable. The true genome size of bread wheat has been estimated by flow cytometry to be close to 16 Gb [5]; based on this estimate our assembly contains 96% of the genome sequence.

Results

To create the wheat genome assembly, we generated two extremely large primary data sets. The first data set consisted of 7.06 billion Illumina reads containing approximately 1 trillion bases of DNA. The Illumina reads were 150-bp, paired reads from short DNA fragments, averaging 400 bp in length. Using an estimated genome size of 15.3 Gbp, this represented 65-fold coverage of the genome. The second data set used Pacific Biosciences single-molecule (SMRT) technology to generate 55.5 million reads with an average read length just under 10,000 bp, containing a total of 545 billion bases of DNA, representing 36-fold coverage of the genome. All reads were generated from the Chinese spring variety (CS42, accession Dv418) of *T. aestivum*, the same variety as used in earlier attempts to sequence the genome.

MaSuRCA assembly

To create the initial assembly, Triticum 1.0, we ran the MaSuRCA assembler (v. 3.2.1) on the full data set of Illumina and PacBio reads (MaSuRCA, RRID:SCR_010691). The first major step was the creation of super-reads [6] from the Illumina reads. Super-reads are highly accurate and longer than the original reads, and because they are much fewer in number, they provide a means to greatly compress the original data. This step generated 95.7 million super-reads with a total length of 31 Gb, a mean size of 324 bp and an N50 size of 474 bp (i.e., half of the total super-read sequence was contained in super-reads of 474 bp or longer). The super-reads provided a 32-fold compression of the original Illumina data.

Next we created *mega-reads* by using the super-reads to tile the PacBio reads, effectively replacing most PacBio reads (which have an average error rate of ~15%) with much more accurate sequences [7]. Most PacBio reads were converted into a single mega-read, but in some

cases a given PacBio read yielded two or more (shorter) mega-reads. In total we created 57,020,767 mega-reads with a mean length of 4,876 bp and an N50 length of 8,427 bp. The total length of the mega-reads was 278 Gb, representing about 18X genome coverage. As part of this step, we also created synthetic mate pairs; these link together two mega-reads when the pair of mega-reads originates from a single PacBio read. We generated these pairs by extracting 400 bp from opposite ends of each pair of consecutive mega-reads corresponding to a given PacBio read. This resulted in 23.45 million pairs of 400 bp reads, totalling 18.75 Gb.

Construction of super-reads and mega-reads required approximately 100,000 CPU hours, of which 95% was spent in the mega-reads step. By using large multi-core computers to run these steps in parallel, these steps took 1.5 months of elapsed (wall clock) time. The peak memory (RAM) usage was 1.2 terabytes.

We then assembled the mega-reads and the synthetic pairs using the Celera Assembler [8] (v8.3), which was modified to work with our parallel job scheduling system. (The modified software is available at ftp://ftp.ccb.jhu.edu/pub/dpuiu/OTHER/SLURM/runCA.) The CA assembly process required many iterations of the overlapping, error correction, and contig construction steps, and it was extremely time consuming, even with the many optimizations that have been incorporated in this assembler in recent releases. The total CPU time was ~470,000 CPU hours (53.7 *years*), which was only made feasible by running it on a grid with thousands of jobs running in parallel (the maximum number was 3,320) for some of the major steps. The total elapsed time was just over 5 months. When combined with the earlier steps, the entire assembly process took 6.5

months. The resulting assembly, labelled Triticum 1.0, contained 17.046 Gb in 829,839 contigs, with an N50 contig size of 76,267 bp and an N50 scaffold size of 101,195 bp (**Table 1**).

Table 1. Assembly statistics for each of the assemblies of *Triticum aestivum* constructed as described in the text. To enable fair comparisons, all N50 sizes are computed using an estimated genome size of 15.34 Gb.

Assembly	Element type	Number	Total size (bp)	Average size (bp)	N50 size (bp)
Triticum 1.0	contigs	829,839	17,045,571,778	20,541	76,267
	scaffolds>2Kb	576,137	16,889,295,941	29,314	101,195
Triticum 2.0	contigs	375,328	14,395,027,822	38,353	75,599
	scaffolds>2Kb	252,501	14,412,484,332	57,078	100,805
FALCON Trit 1.0	contigs	97,809	12,939,100,857	132,289	215,314
Triticum 3.0	contigs	279,439	15,343,711,528	54,908	232,613
Triticum 3.1	contigs	279,439	15,344,693,583	54,912	232,659

Next, in order to detect and remove redundant regions of the assembly, we aligned the assembly against itself using the nucmer program from the MUMmer package [9]. We identified and excluded scaffolds that were completely contained in and ≥96% identical to other scaffolds. After this de-duplication procedure, the reduced assembly, Triticum 2.0, contained 14.40 Gbp in 375,328 contigs with an N50 contig size of 75,599 bp, with scaffolds spanning 14.45 Gbp and an N50 scaffold size of 100,805 bp (**Table 1**).

FALCON assembly

Independently of the MaSuRCA assembly, we assembled the PacBio data alone using the FALCON assembler [10], followed by polishing with the Arrow program, which substantially improves the consensus accuracy. FALCON implements a hierarchical assembly approach; the initial step is to error correct long reads by aligning all reads to a subset of the longest reads. Given the relatively low raw read coverage (36X), we used a long-read cutoff of 1 Kb, generating 11X coverage of error-corrected reads with an N50 size of 16 Kb. Error correction and assembly of the corrected reads was completed using ~150,000 CPU hours, which took ~3

weeks on a 16-node cluster. The contigs output from FALCON require further polishing, which involves realignment of raw reads and calculation of a new consensus [11]. For the polishing step, we used Pacbio's resequencing pipeline from the SMRT Analysis package (https://github.com/PacificBiosciences/SMRT-Link) after first splitting the assembled contigs into <4 Gbp chunks (a limit of the aligner). Polishing required an additional ~160,000 CPU hours, for a total of 310,000 CPU hours and 6 weeks elapsed (wall clock) time.

These steps produced an assembly, designated FALCON Trit 1.0, containing 12.94 Gbp in 97,809 contigs with a mean size of 132,289 and an N50 size of 215,314 bp (**Table 1**).

Merged assembly

The contigs from the FALCON assembly were larger than those from the MaSuRCA assembly; however, the total size of the assembly was 1.5 Gbp smaller. To capture the advantages of both assemblies, we merged them as follows. We aligned the contigs (not scaffolds) from the two

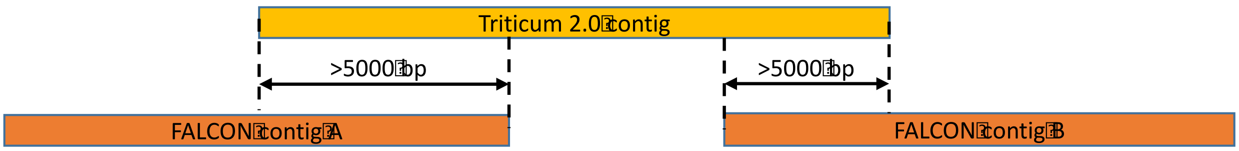

Figure 1. Illustration of the merging process for the Triticum 2.0 and FALCON Trit 1.0 assemblies. If two contigs A and B from the FALCON assembly overlapped a Triticum 2.0 contig by at least 5000 bp, then A and B were merged together, using the Triticum 2.0 contig to fill the gap.

assemblies using MUMmer 4.0 [9] and extracted all pairwise best matches. We then merged each pair of FALCON contigs when they overlapped a single Triticum 2.0 contig by at least 5000 bp, with Triticum 2.0 sequence filling the gap (see **Figure 1**).

After merging and extending the FALCON contigs, we then identified all MaSuRCA scaffolds that were not contained in the longer FALCON contigs, and added these to the new assembly. The resulting merged assembly, Triticum 3.0, contains 15,343,750,409 bp in 279,529 contigs, with a contig N50 size of 232,613 bp (**Table 1**). The longest contig is 4,510,883 bp. The assembly contains no unknown (N) bases.

Genome complexity

As described above, previous attempts to assemble the hexaploid wheat genome were stymied because of its exceptionally high repetitiveness, but until now we had no reliable way to quantify how repetitive the genome truly is. To answer this question with a precise metric, we computed the k-mer uniqueness ratio, a metric defined earlier as a way to capture repetitiveness that reflects the difficulty of assembly [12]. This ratio is defined as the percentage of a genome that is covered by unique sequences of length *k* or longer. If, for example, 90% of a genome is comprised of unique 50-mers, then one might expect that 90% of that genome could be assembled using accurate (low-error-rate) reads that were longer than 50 bp.

With the Triticum 3.0 assembly in hand, we computed the k-mer uniqueness ratio for wheat and compared it to several other plant and animal genomes, as shown in **Figure 2**. As the figure illustrates, for any value of k, a much smaller percentage of the wheat genome is covered by unique k-mers than other plant or animal genomes, with the exception of *Ae. tauschii*, which as expected (because it is near-identical to the D genome of hexaploid *T. aestivum*) is only slightly less repetitive. For example, only 44% of the 64-mers in the wheat genome are unique, as contrasted with 90% of the 64-mers in cow and 81% of the 64-mers in rice. This analysis demonstrates that in order to obtain an assembly covering most of the wheat genome, particularly if the algorithm relies on de Bruijn graphs, much longer reads will be required. Our

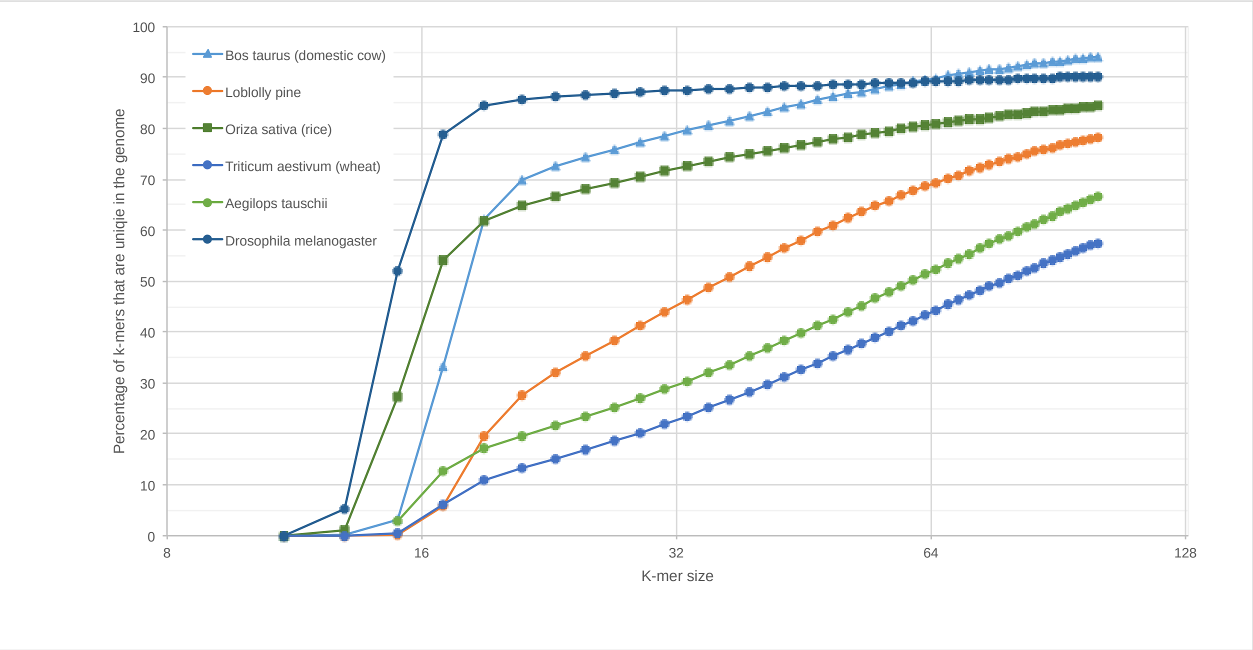

Figure 2. K-mer uniqueness ratios for the wheat genome (*Triticum aestivum*) compared to the cow, fruit fly, rice, loblolly pine, and *Ae. tauschii* genomes. The plot shows the percentage of each genome that is covered (y-axis) by unique sequences of length k, for various values of k (x-axis).

sequencing strategy, by using deep coverage in very long PacBio reads coupled with highly accurate Illumina reads, was able to produce the long, accurate reads required to assemble this very complex genome.

Identifying the wheat D genome

T. aestivum is a hexaploid plant with three diploid ancestors, one of which is *Aegilops tauschii*, commonly known as goat grass. *Ae. tauschii* itself is a highly repetitive genome that has resisted attempts at assembly, but we recently published a highly contiguous draft assembly (Aet_MR 1.0) using a similar strategy to the one used for wheat, a combination of PacBio and Illumina sequences [7]. *T. aestivum*'s hexaploid composition is typically represented as AABBDD, where the D genome was contributed by an ancestor of *Ae. tauschii*. The hexaploidization event occurred very recently, approximately 8,000 years ago, when *Ae. tauschii* spontaneously hybridized with a tetraploid wheat species, *Triticum turgidum* [13].

Because this event was so recent, the wheat D genome and *Ae. tauschii* are highly similar, much closer to one another than the D genome is to either the A or B genomes. We used this similarity to identify the D genome components of our assembly by aligning the *Ae. tauschii* contigs in Aet_MR 1.0 to Triticum 3.0. We used the nucmer program [9] to identify all alignments representing best matches between Triticum 3.0 and Aet_MR 1.0 with a minimum identity of 97%. The vast majority of the two genomes are >99% identical, making this filtering process relatively straightforward.

After filtering, we identified 50,101 contigs with a total length of 4,179,762,575 bp from Triticum 3.0 that aligned to *Ae. tauschii*. We separated these D genome contigs from Triticum 3.0 and provided them as the first release of the wheat D genome, which we have named TriticumD 1.0. The N50 size of these contigs is 224,953 bp, using a genome size estimate of 4.18

Gb for wheat D. The total size of 4.18 Gb corresponds closely to the 4.33 Gb in the recently published *Ae. tauschii* (Aet_MR 1.0) assembly [7].

We also ran the alignments in the other direction, aligning all of Aet_MR 1.0 to TriticumD 1.0, and found that 99.8% of the *Ae. tauschii* assembly matches TriticumD; only 8.96 Mb failed to align. The overall mapping is complex; although most of the *Ae. tauschii* and wheat D genomes align in a 1-to-1 mapping, many scaffolds align in a many-to-one or one-to-many arrangement. Thus the additional 150 Mb in *Ae. tauschii* appears to be due to gain/loss of repeats rather than loss of unique sequence from wheat D.

Assembly quality and completeness.

Assessing the quality of an assembly is challenging, especially when the previous assemblies are so much more fragmented, as they are in the case of *T. aestivum*. However, the very high-fidelity alignments between Triticum 3.0 and the published *Ae. tauschii* genome, at over 99% identity, provide strong support for its accuracy. We found no large-scale structural disagreements between the assemblies, other than the many-to-one mappings for some of the scaffolds. These could indicate that one assembly has over-collapsed a repeat, but they could also indicate a true polymorphism; we do not have sufficient data to distinguish these possibilities. The fact that 99.8% of *Ae. tauschii* aligns to Triticum 3.0 supports the hypothesis that the assembly is largely complete as well.

As a further evaluation of assembly quality, we aligned 19,401 BAC ends from the wheat chromosome 3B-specific BAC library, TA3B (NCBI BioSample SAMN001187987) [14] to all contigs in Triticum 3.1. 18,465 BAC ends aligned, of which 2,739 pairs aligned to the same contig. Of these 2,739 pairs, 2,709 (99%) aligned in the correct orientation with a distance consistent with the mean size for the library. In no case did a pair of BAC ends align to a single contig in the wrong orientation. Out of all BACs where the ends aligned to different contigs, only 282 had one BAC end aligning sufficiently far from a contig's end to permit the other BAC end to align to the same contig; these could represent mis-assembled contigs, but they could also be explained by unusually long BACs or alignment artifacts.

We used BUSCO (version 3.0.2)(BUSCO, RRID:SCR_015008) [15] to assess the completeness of the Triticum 3.1 assembly based on the presence of the single-copy orthologs from the OrthoDB (v9.1) [16] database(OrthoDB, RRID:SCR_011980). We found that 1415 out of 1440 BUSCO genes are present and complete in the Triticum 3.1 assembly, of which 161 ae single-copy and 1254 are in multiple copies. The large number of duplicated genes is likely due to the polyploidy of the genome. Only 4 BUSCO genes are fragmented and 21 are missing. We ran the same analysis on most complete published bread wheat assembly, TGACv1 [3], and found that it contains 1411 complete BUSCO genes (very slightly fewer than Triticum 3.1), of which 126 are single-copy, 1285 are multiple-copy, 8 are fragmented and 21 are missing.

Re-polishing to create Triticum 3.1

Finally, we used an independent set of Illumina 250-bp reads from an earlier study [3] to measure the quality of the consensus sequence. We used the KAT program [17] to count all 31-mers in each assembly and compare these counts to the 31-mers in the read data. Because the

read data here represented 30-fold coverage of the genome, 31-mers that occur approximately 30 times should represent unique sequences; i.e., they are expected to occur exactly once in the assembly.

The KAT analysis revealed that the FALCON Trit 1.0 assembly was missing a relatively large number of 31-mers that occurred in the reads (**Figure 3**), while the MaSuRCA-derived Triticum 2.0 assembly was missing far fewer of these 31-mers. The Triticum 3.0 assembly, which used the polished FALCON contigs for most of its consensus sequence, was also missing many 31-mers. The mostly likely explanation for this effect is that the polishing process over-corrected by replacing some 31-mers with near-identical ones. This would have the effect of creating an excess of 31-mers that occur exactly twice in the assembly, although their coverage indicated that they should occur once. The KAT analysis confirmed this expectation.

Because Triticum 2.0 had far fewer missing 31-mers, and because it created its consensus from mega-reads whose sequence was based on Illumina data, we re-polished Triticum 3.0 by aligning it to Triticum 2.0, extracting the mutual best matches, and then using the 2.0 sequence as the final consensus. This allowed us to replace about 98% of the Falcon consensus in the 3.0 assembly by the higher-quality MaSuRCA consensus. The resulting assembly, Triticum 3.1, has exactly the same number of contigs and scaffolds (**Table 1**) but has an improved overall consensus, containing more of the true 31-mers (**Figure 3**). Because of changes in the consensus sequence, the 3.1 assembly is very slightly larger as well. To evaluate the possibility of further improvements, we analysed the 31-mer spectra of both FALCON Trit 1.0 and Triticum 2.0 as a single sequence set. We found that this almost completely eliminated the missing 31-mers

(**Figure 3**), illustrating that further improvements in the consensus are possible and are planned for future assembly releases.

Discussion

In 2004, an international consortium determined that whole-genome shotgun (WGS) sequencing

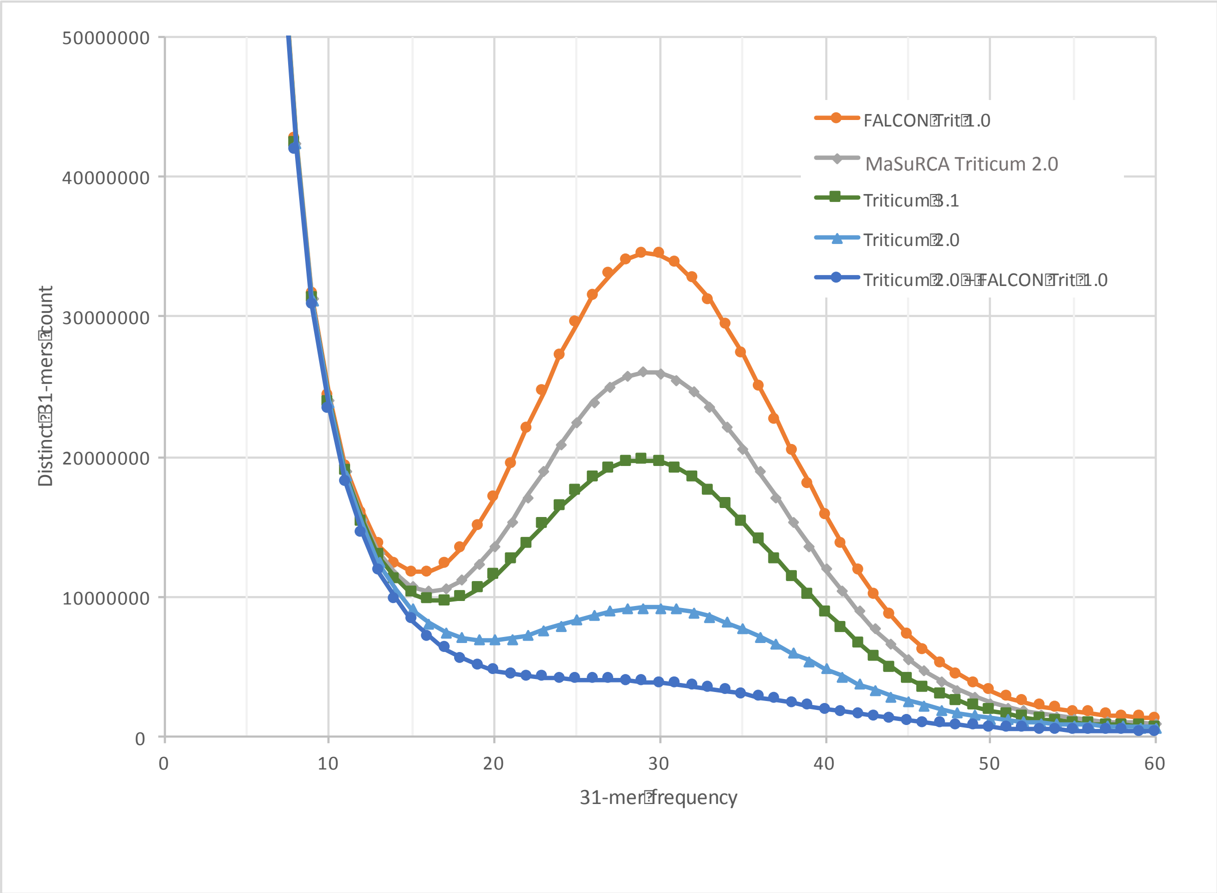

Figure 3. Missing 31-mers in the different assemblies of *Triticum aestivum*. Using the Illumina read data from a previously published assembly of the same genome, we counted all 31-mers in the reads, and then plotted how many of these 31-mers are missing from each assembly. The x-axis shows how often the k-mers occur in the reads. The y-axis shows how many distinct k-mers are missing from each assembly. The FALCON Trit 1.0 assembly had the largest number of missing k-mers, while the MaSuRCA-driven Triticum 2.0 assembly had the fewest.

of hexaploid wheat was simply too difficult, "mainly because of the large size and highly repetitive nature of the wheat genome" [18]. The consortium instead determined that the

chromosome-by-chromosome approach would be more effective. This strategy, which was far slower and more costly than WGS sequencing, produced a genome assembly that was highly fragmented and that contained only 10.2 Gb [2].

The assembly described here is the first to successfully reconstruct essentially all of the hexaploid wheat genome, *Triticum aestivum*, and to produce relatively large contiguous sequences. The final assembly contains 15,344,693,583 bp with an N50 contig size of 232,659 bp. The previous chromosome-based assembly was not only much smaller overall, but it had average contig sizes approximately 50 times smaller [2]. A recent whole-genome assembly based on deep Illumina sequencing contained 2,726,911 contigs spanning 12,658,314,504 bp and had a contig N50 size of 9731 bp [3]. Compared to Triticum 3.0, that assembly is 2.69 Gb smaller, and its contigs are 24 times smaller. (Note that in order to provide a fair comparison, all N50 sizes reported here are based on the same 15.34 Gb total genome size.)

Why did previous attempts to assemble *T. aestivum* produce a result that was billions of nucleotides shorter than the true genome size? The most likely explanation is that the repetitive sequences, which cover some 90% of the genome [4, 18], are so similar to one another that genome assembly programs cannot avoid collapsing them together. This is a well-known problem for genome assembly, particularly when using the short reads produced by next-generation sequencing technologies. If the differences between repeats occur at a lower rate than sequencing errors, then assemblers cannot distinguish them. The result is an assembly that is both highly fragmented and too short. The same phenomenon can be seen in attempts to assemble *Ae. tauschii*. from short reads. An assembly of that genome using Illumina and 454

sequencing data, contained only 2.69 Gb and had an N50 contig size of just 2.1 Kb [13]. A hybrid assembly using both Illumina and PacBio data, reported by our group early in 2017, produced an assembly of 4.33 Gb, closely matching the estimated genome size, with a contig N50 size of 487 Kb [7].

The key factor in producing a true draft assembly for this exceptionally repetitive genome was the use of very long reads, averaging just under 10,000 bp each, which were required to span the long, ubiquitous repeats in the wheat genome. Deep coverage in these reads (36X, or 545 Gb of raw sequence) coupled with even deeper coverage (65X) in low-error-rate short reads, allowed us to produce a highly accurate and highly contiguous consensus assembly. The massive data set, over 1.5 trillion bases, also required an unprecedented amount of computing power to assemble, and its completion would not have been possible without the availability of very large parallel computing grids. All together, the various assembly steps took 880,000 CPU hours, or just over 100 CPU years. An important technical note is that the computational cost was not simply a function of genome size, but more critically a function of its repetitiveness. The presence of large numbers of unusually long exact and near-exact repeats (Figure 2) means that all of these sequences overlap one another, leading to a quadratic increase in the number of sequence alignments that an assembler must consider.

Finally, ours is the first assembly to cleanly separate the D genome component from the A and B genomes of hexaploid wheat by aligning this assembly to the draft genome of *Aegilops tauschii*, the progenitor of the wheat D genome. This separation was possible because *Ae. tauschii* is much

closer to wheat D, having diverged approximately 8,000 years ago [18], than either genome is to wheat A or B.

The wheat genome presented here provides, for the first time, a near-complete substrate for future studies of this important food crop. Previous efforts to annotate the genome have been hampered by the absence of a large proportion of the genome itself, making inferences about missing genes or gene families difficult, and also by the highly fragmented nature of previous assemblies, which had average contig sizes under 10 Kb. With over half of the genome now contained in contigs longer than 232 Kb, the Triticum 3.0 assembly will contain many more genes within single contigs, greatly aiding future efforts, which are already under way, to study its gene content, evolution, and relationship to other plant species.

Availability of data. The Triticum project data have been deposited at the National Center for Biotechnology Information (NCBI) under BioProject PRJNA392179. The assembly has been deposited at DDBJ/ENA/GenBank under the accession NMPL00000000. The version described in this paper is version NMPL01000000. The PacBio and Illumina reads are available under the same BioProject. The TriticumD 1.0 contigs are available separately at ftp://ftp.ccb.jhu.edu/pub/data/Triticum_aestivum/Wheat_D_genome. The preliminary assemblies described in the paper and other supporting files are also available in the *GigaScience* GigaDB database [19].

Competing interests statement. Two of the authors are employees of Pacific Biosciences. None of the other authors have any competing or conflicting interests.

Acknowledgements

The authors wish to thank Jan Dvořák for helpful comments on the manuscript. We also acknowledge the support of the Johns Hopkins University large-scale computing center, MARCC, which provided invaluable computing resources. This work was supported in part by the U.S. National Science Foundation under grants IOS-1238231 and IOS-1444893, and by the U.S. National Institutes of Health under grant R01 HG006677.

References

1. Brenchley R., M. Spannagl, M. Pfeifer, G.L. Barker, R. D'Amore, A.M. Allen, ..., and N. Hall. Analysis of the bread wheat genome using whole-genome shotgun sequencing. *Nature*, 2012. **491**(7426): 705-10.
2. International Wheat Genome Sequencing C. A chromosome-based draft sequence of the hexaploid bread wheat (*Triticum aestivum*) genome. *Science*, 2014. **345**(6194): 1251788.
3. Clavijo B.J., L. Venturini, C. Schudoma, G.G. Accinelli, G. Kaithakottil, J. Wright, ..., and M.D. Clark. An improved assembly and annotation of the allohexaploid wheat genome identifies complete families of agronomic genes and provides genomic evidence for chromosomal translocations. *Genome Res*, 2017. **27**(5): 885-896.
4. Li W., P. Zhang, J.P. Fellers, B. Friebe, and B.S. Gill. Sequence composition, organization, and evolution of the core Triticeae genome. *Plant J*, 2004. **40**(4): 500-11.
5. Arumuganathan K. and E.D. Earle. Nuclear DNA content of some important plant species. *Plant Molecular Biology Reporter*, 1991. **9**(3): 208-218.
6. Zimin A.V., G. Marcais, D. Puiu, M. Roberts, S.L. Salzberg, and J.A. Yorke. The MaSuRCA genome assembler. *Bioinformatics*, 2013. **29**(21): 2669-77.
7. Zimin A.V., D. Puiu, M.C. Luo, T. Zhu, S. Koren, G. Marcais, ..., and S.L. Salzberg. Hybrid assembly of the large and highly repetitive genome of Aegilops tauschii, a progenitor of bread wheat, with the MaSuRCA mega-reads algorithm. *Genome Res*, 2017. **27**(5): 787-792.
8. Myers E.W., G.G. Sutton, A.L. Delcher, I.M. Dew, D.P. Fasulo, M.J. Flanigan, ..., and J.C. Venter. A whole-genome assembly of Drosophila. *Science*, 2000. **287**(5461): 2196-204.
9. Kurtz S., A. Phillippy, A.L. Delcher, M. Smoot, M. Shumway, C. Antonescu, and S.L. Salzberg. Versatile and open software for comparing large genomes. *Genome Biol*, 2004. **5**(2): R12.

10. Chin C.S., P. Peluso, F.J. Sedlazeck, M. Nattestad, G.T. Concepcion, A. Clum, ..., and M.C. Schatz. Phased diploid genome assembly with single-molecule real-time sequencing. *Nat Methods*, 2016. **13**(12): 1050-1054.
11. Chin C.S., D.H. Alexander, P. Marks, A.A. Klammer, J. Drake, C. Heiner, ..., and J. Korlach. Nonhybrid, finished microbial genome assemblies from long-read SMRT sequencing data. *Nat Methods*, 2013. **10**(6): 563-9.
12. Schatz M.C., A.L. Delcher, and S.L. Salzberg. Assembly of large genomes using second-generation sequencing. *Genome research*, 2010. **20**(9): 1165-73.
13. Jia J., S. Zhao, X. Kong, Y. Li, G. Zhao, W. He, ..., and J. Wang. *Aegilops tauschii* draft genome sequence reveals a gene repertoire for wheat adaptation. *Nature*, 2013. **496**(7443): 91-5.
14. Safar J., J. Bartos, J. Janda, A. Bellec, M. Kubalakova, M. Valarik, ..., and B. Chalhoub. Dissecting large and complex genomes: flow sorting and BAC cloning of individual chromosomes from bread wheat. *Plant J*, 2004. **39**(6): 960-8.
15. Simao F.A., R.M. Waterhouse, P. Ioannidis, E.V. Kriventseva, and E.M. Zdobnov. BUSCO: assessing genome assembly and annotation completeness with single-copy orthologs. *Bioinformatics*, 2015. **31**(19): 3210-2.
16. Zdobnov E.M., F. Tegenfeldt, D. Kuznetsov, R.M. Waterhouse, F.A. Simao, P. Ioannidis, ..., and E.V. Kriventseva. OrthoDB v9.1: cataloging evolutionary and functional annotations for animal, fungal, plant, archaeal, bacterial and viral orthologs. *Nucleic Acids Res*, 2017. **45**(D1): D744-D749.
17. Mapleson D., G. Garcia Accinelli, G. Kettleborough, J. Wright, and B.J. Clavijo. KAT: a K-mer analysis toolkit to quality control NGS datasets and genome assemblies. *Bioinformatics*, 2017. **33**(4): 574-576.
18. Gill B.S., R. Appels, A.M. Botha-Oberholster, C.R. Buell, J.L. Bennetzen, B. Chalhoub, ..., and T. Sasaki. A workshop report on wheat genome sequencing: International Genome Research on Wheat Consortium. *Genetics*, 2004. **168**(2): 1087-96.
19. Zimin, A, V; Puiu, D; Hall, R; Kingan, S; Clavijo, B; Salzberg, S, L (2017): Supporting data for the near-complete assembly of the hexaploid bread wheat genome, Triticum aestivum GigaScience Database. http://dx.doi.org/10.5524/100356

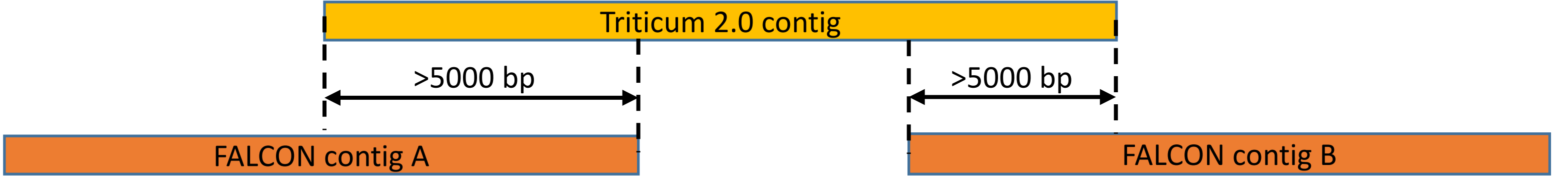
Triticum 2.0 contig
>5000 bp
>5000 bp
FALCON contig A
FALCON contig B

Figure 2. K-mer uniqueness ratios for the wheat genome (Triticum aestivum) compared to the cow, fruit fly, rice, loblolly pine, and Ae. tauschii genomes.

Click here to download Figure Figure2-kmer-uniqueness.pdf

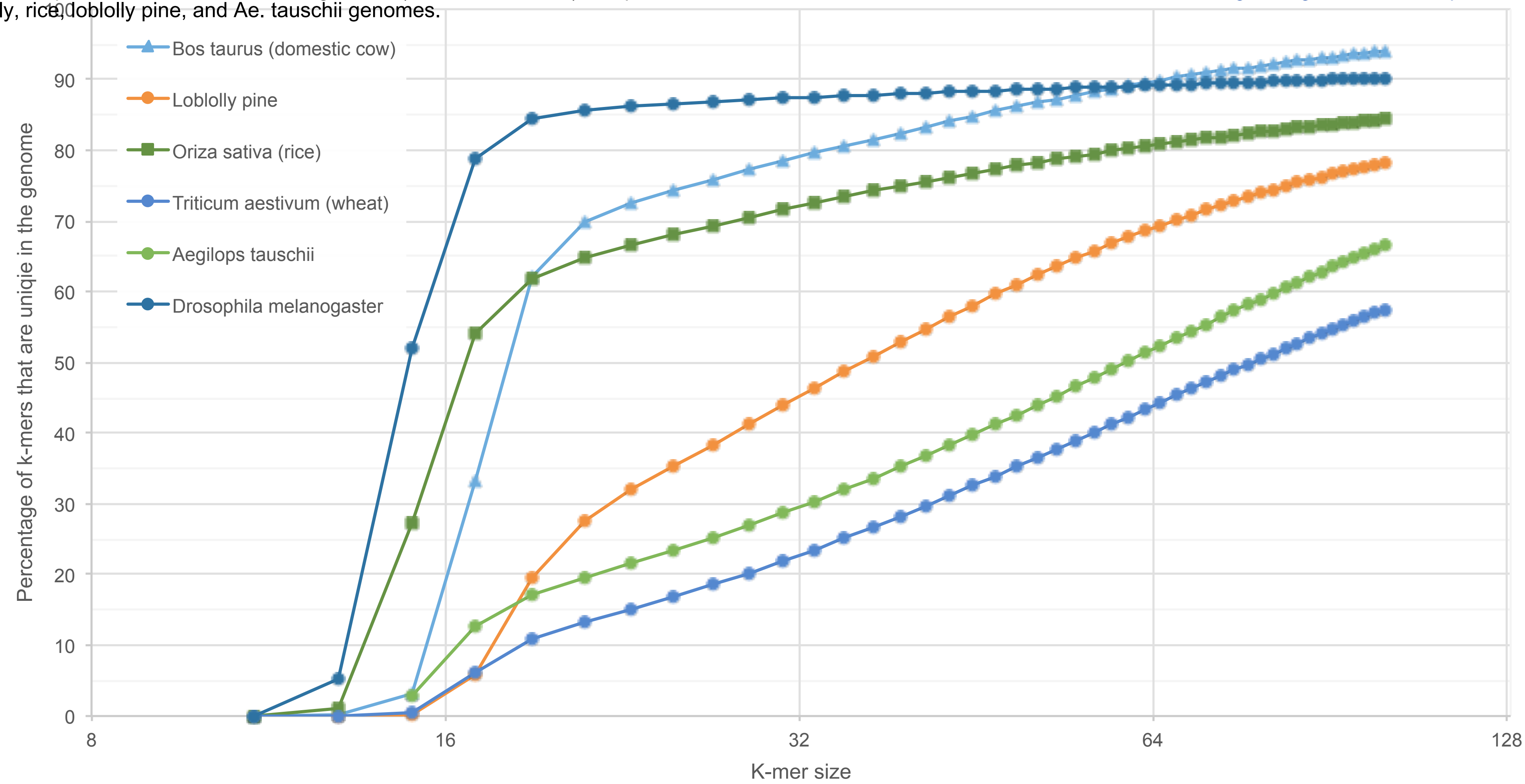

Figure 3. Missing 31-mers in the different assemblies of Triticum aestivum.

Click here to download Figure Absent kmers.pdf

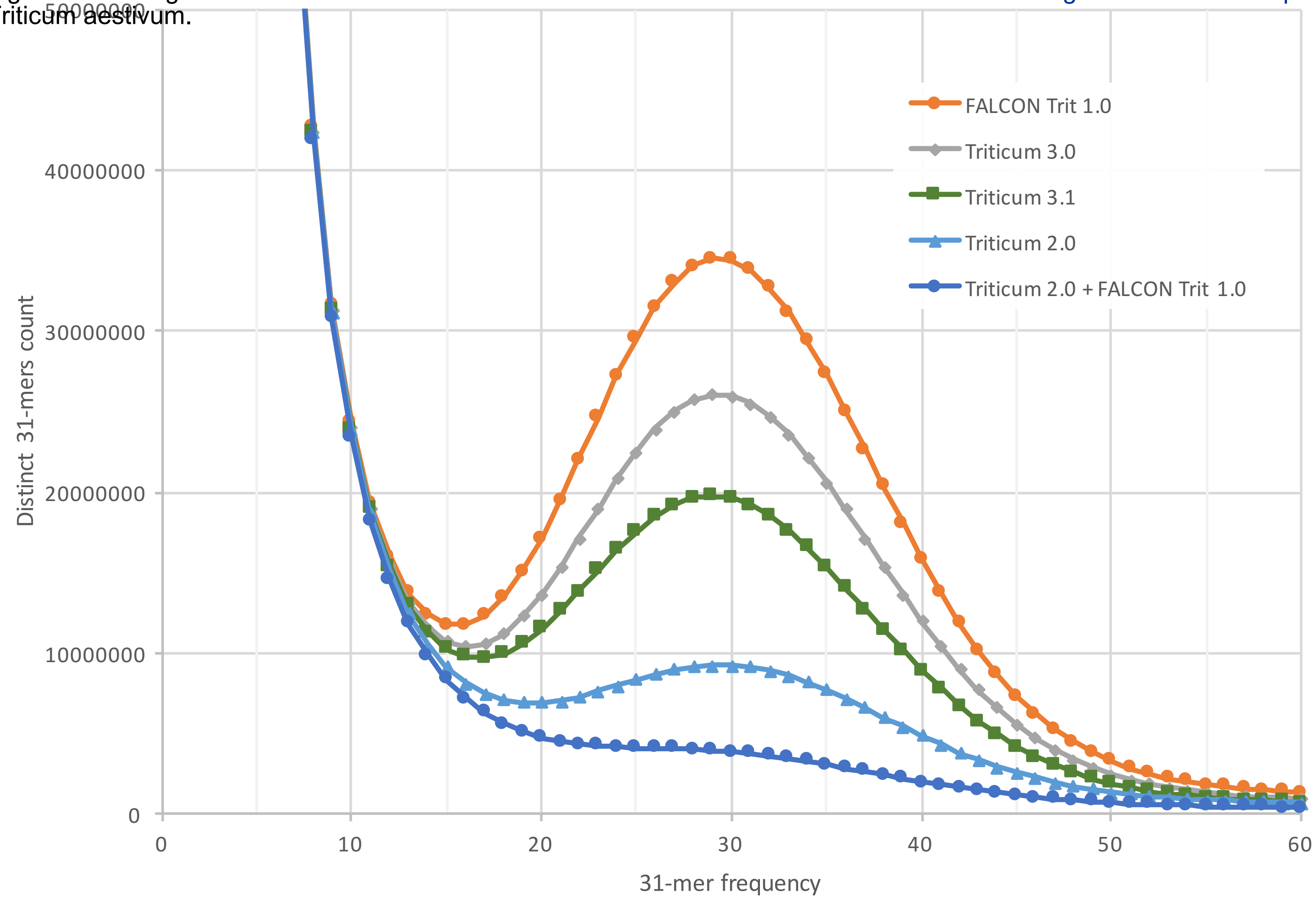

 Click here to download Personal Cover Response-to-reviewers-82517.docx

Dear Editors,
We have responded to all of the reviewers' comments in our revised manuscript. We thank the reviewers for their comments which helped to improve the presentation.

First we have a meta-comment. Because the reviewers are not anonymous, we discovered that all three reviewers are either current or past members of the IWGSC (Internatl Wheat Genome Sequencing Consortium). The IWGSC has a competing assembly of hexaploid wheat that they have been talking about at meetings, but that is not publicly available. They have stated that they are working on a paper, and this paper is clearly in direct competition with ours. Several of the reviewers' comments refer to this non-public genome and to their previous publications with the IWGSC. Thus as direct competitors, all 3 reviewers have a strong conflict of interest and should not have reviewed our paper; we believe they should have self-identified as being in conflict and recused themselves. However, they did not do so.

Nonetheless, we did the best we could to address all comments. Because of this strong conflict, though, and because these reviewers might be strongly motivated to delay publication of our paper, we would ask that the GigaScience editors read our responses and make a final decision without relying on the reviewers' further comments. We are very concerned that the reviewers will simply make more requests for changes and will attempt to delay our paper indefinitely, or at least until their own paper can appear.

We believe we have addressed the legitimate concerns adequately, as we explain below. We list each of the comments followed by our responses, indicating how we changed the manuscript in each case. Note that we omitted those comments not requiring a response. For clarity, the reviewers' comments are shown in italics.
Sincerely,
Steven Salzberg and Aleksey Zimin (on behalf of all the authors)
==

Reviewer #1: *In their paper, Zimin et al. present a new assembly for the wheat cultivar Chinese Spring based on a large amount of PacBio and Illumina reads. They state that this is the most complete wheat genome assembly to date (15.34 Gbp), and this makes it a potentially very valuable resource for researchers. The authors then use this assembly to align with an older Aegilops tauschii genome to separate candidate D-genome scaffolds.*
While there is no biology in this paper, it is purely technical, the advance is still of interest and the manuscript is a good fit for GigaScience as it has published quite a few technical genome assembly papers. It is very interesting that the polishing step in v3.0 removed many unique k-mers that were then reintroduced by alignment with v2.0 to generate v3.1. In their paper the authors should mention that the assembly does not include a single unknown base (N).

We thank the reviewer for these positive comments. We added a sentence to the abstract stating: "Our assembly contains no unknown (N) bases."

Some of the text is over simplistic, saying that all other attempts at assembly have failed does

not take into account of the aims of those projects which were often just to obtain the unique and low copy regions. The authors repeatedly say that this is the best assembly to date but ignore the available NRGene Chinese Spring assembly. I understand that this has not been published and so a direct comparison cannot be made, but the authors should at least acknowledge that NRGene assembly is available and compare general statistics. Saying that the wheat genome 'resisted efforts' suggests it did this deliberately. Suggesting that long reads = good, short reads = bad is again over simplistic. Repeats can be assembled with short reads when there is read pair information which is another common approach, the authors should acknowledge this and also comment on the read quality difference between long and short read sequencing. The authors claim 6 copies of each chromosome, but they should clarify that due to homozygosity, they only assemble 3 copies, A, B and D.

We rephrased the text describing the previous efforts. Instead of saying that "Multiple past attempts to assemble the genome have failed," we revised the abstract to say:
"Multiple past attempts to assemble the genome have produced assemblies that were well short of the estimated genome size."

Note that the phrase "resisted efforts to sequence it" is a common English construction, and does not mean the genome did this deliberately. This expression accurately reflects our message that the genome has been very challenging to sequence and assemble. However, we did rephrase several other statements in the introduction, as follows. To explain more clearly that the assembly contains 3 (rather than 6) copies of each chromosome, we added the following:
"All data for this assembly was generated from the Chinese spring variety (CS42, accession Dv418) of *T. aestivum*, which is highly inbred and thus nearly haploid, effectively reducing the number of copies of each chromosome from six to three."

Note that nowhere do we state that "that long reads = good, short reads = bad" as the reviewer put it. Indeed, in our previous papers we have described the benefits of various paired-end libraries, but this paper is not a review of assembly strategies, so we preferred not to engage in a lengthy digression to explain this. (One of us is a co-author of a 2010 review describing the challenges of short read sequencing – Schatz et al 2010 – which we reference in the text here.)

The reviewer mentions the "available NRGene Chinese Spring assembly" and asks for comparisons to it. The NRGene assembly is not freely or publicly available, and we do not have it. Obtaining it requires registration and signing a restrictive agreement with the IWGSC on data usage, which we will not do on principle. (Even if we did, the restrictive agreement might prevent us from including any detailed comparisons in our paper.) We also note that this genome assembly has not been published and therefore no metrics or statistics are available to which we can compare our results.

The authors use a predicted genome size of 15.3 Gbp which is on the small size for wheat genome predictions. The authors should justify the use of this number. The differences between Ae. tauschii and the bread wheat genome are suggested to be technical errors or loss or gain of

repeats. The authors should read the recent wheat pangenome paper which shows that you would expect gene presence absence variation between varieties and hence also between the diploid and polyploid.

We revised the paper to clarify (in the text and in the Table 1 caption) that we use 15.34 Gb for computing the N50 statistics for comparison of various assemblies. The computation of the N50 statistics depends critically on the assumed genome size, not the assembly size. As long as the same reasonable estimate of the genome size is used for all assemblies, contiguity comparisons are valid. We added text at the end of the introduction to make it clear that the true genome size is likely a bit larger. We now cite the flow cytometry estimate of 16Gb from (Arumuganathan and Earle, 1991). Our revised text says:
"In this paper we use 15.34 billion bases as the genome assembly size for computing the N50 statistics of different assemblies, in order to make these statistics comparable. The true genome size of bread wheat has been estimated by flow cytometry to be close to 16 Gb [5]; based on this estimate our assembly contains 96% of the genome sequence."

The main issue I have with the manuscript is the lack of quality control. The authors make statements saying that they are the first to 'reconstruct essentially all of the hexaploid wheat genome' but the assembly lacks the majority of quality control required for genome publications. Running BUSCO should be relatively straight forward and would provide a direct comparison between this and all the other assemblies (including the NRGene one and the reassembled chromosome arms from Montenegro et al. (2017)). Are all the genes identified in the previous assemblies in this one? Given that this assembly is based on long reads with low accuracy, what is the bp similarity between related portions of each of the assemblies?

We had not used BUSCO because earlier versions had failed (crashed) when we ran them on large plant genomes, but prompted by the reviewer, we downloaded the latest version (3.0.2), and it appears to work now. We then used BUSCO to compare the single copy orthologous gene content of our assembly to the TGACv1 wheat assembly, which is the best assembly published to date. The results show that our assembly completeness looks excellent, and is very slightly better than TGACv1. We added the following text to the manuscript in the Assembly quality sub-section, which we renamed to "Assembly quality and completeness":

"We used BUSCO (version 3.0.2) [14] to assess the completeness of the Triticum 3.1 assembly based on the presence of the single-copy orthologs from the OrthoDB (v9.1) [15] database. We found that 1415 out of 1440 BUSCO genes are present and complete in the Triticum 3.1 assembly, of which 161 ae single-copy and 1254 are in multiple copies. The large number of duplicated genes is likely due to the polyploidy of the genome. Only 4 BUSCO genes are fragmented and 21 are missing. We ran the same analysis on most complete published bread wheat assembly, TGACv1 [3], and found that it contains 1411 complete BUSCO genes (very slightly fewer than Triticum 3.1), of which 126 are single-copy, 1285 are multiple-copy, 8 are fragmented and 21 are missing."

Some specific issues:

Some of the language should be more precise and specific, eg. Line 61 'dramatically' better or line 62 'essentially the entire length of the genome' - this is not demonstrated. Line 89 'Most PacBio' and 'some cases', actual numbers here are important. On line 54, 'reads that contain them' should read 'reads that span them'

We revised the text, removing "dramatically better" and using precise numbers as requested. The revised text says:
"By combining these very long reads with highly accurate shorter reads, we have been able to produce an assembly of the wheat genome that is more than ten times more contigous than those produced in any previous attempt. Ours is the first assembly that contains nearly the entire length of the genome, with more than 15.3 billion bases."

It is commendable that the authors expand on the computational needs for this assembly and included some numbers for the CPU hours and walltime used as this will be very valuable data for researchers who need to justify their HPC requests. Can the researchers comment on how many nodes of the cluster the MaSuRCA process used on average and the maximum number of nodes used, if that data is available? The paper states that 'thousands of jobs' were run in parallel, is the exact number still available?

Yes, we checked the logs and found that we ran a maximum of 3320 assembly jobs at a time. In the revised text we added this detail: "thousands of jobs running in parallel (the maximum number was 3,320)"

The paper says that peak memory usage for the mega-reads assembly step was 1.2TB, however, the large memory nodes in MARCC have 1TB of memory. As far as I know MaSuRCA cannot be run in a distributed way so I do not understand where this 1.2TB memory comes from, could the researchers please comment on this?

We needed 1.2TB memory for the mega-reads computation step, which we ran on a single 1.5TB memory computer at the University of Maryland. Most readers will be unaware that MARCC's largest memory node has 1 TB (indeed we were surprised the reviewer knew this), so we didn't add this detail to the text, especially as MARCC might get larger memory nodes soon.

The methods part says that the Celera Assembler was modified to work with the authors' cluster, but the code of that modification does not seem to be available. RunCA already supports SGE clusters, was it just minor modifications on the SGE spec file, or something more complex? Minor changes wouldn't need to be shared, but if the researchers managed to (for example) get RunCA to work with SLURM (as it is used by MARCC) it would be very useful to open source these changes.

We did indeed make minor modifications to the runCA script to let it work with the SLURM scheduler. We added a sentence to the text to explain this, changing this:
"We then assembled the mega-reads and the synthetic pairs using the Celera Assembler [8] (v8.3), which was modified to work with our parallel job scheduling system."

to this:
"We then assembled the mega-reads and the synthetic pairs using the Celera Assembler [8] (v8.3), which was modified to work with our parallel job scheduling system. (The modified software is available at ftp://ftp.ccb.jhu.edu/pub/dpuiu/OTHER/SLURM/runCA.)"

Reviewer #2:

1. Although the authors assert that "full details of the experimental design and statistical methods used [were] given in the Methods section", the manuscript actually does not have a Methods section. The authors should structure their manuscript properly into Introduction, Methods, Results and Discussion sections. The Methods section should contain a description of their pipeline with full details on the software versions and parameter. A flowchart of the assembly process will improve the clarity of the manuscript.

We're not sure what the reviewer is referring to. The manuscript does not contain this phrase, nor does it contain any reference to a "Methods section." We assume this is something from the review form that the reviewer saw. Because this manuscript describes a computational result, we chose to structure it differently (as is common with computational papers) since much of the paper is about the assembly methods. In our view, restructuring the entire paper would not clarify the exposition but more likely would do just the opposite, so we left the structure as is.

2. In l. 19 of the abstract, the authors seem to have used arbitrary thresholds (i.e. assembly length > 15 Gb and N50 > 200 kb) to differentiate between success and failure of assembly efforts. Both (i) the numeric values of these cut-offs and (ii) the arbitrariness of their choice should be stated explicitly before any mention of success or failure is made. Of course, such strong judgmental terms could also simply be omitted.

We removed the statement that previous efforts failed (although one could certainly argue that they did), and instead we revised the abstract to say merely that "Multiple past attempts to assemble the genome have produced assemblies that were well short of the estimated genome size."

3. The better contiguity of the present assembly compared to previous efforts may be due to a higher rate of chimeric scaffolds, i.e. scaffolds combining sequences from physically unlinked regions. I found one such chimera: scaffold '000017F' (1.6 Mb). It has 40 aligned chromosome survey sequence (CSS) contigs originating from 2D (and POPSEQ-anchored to 2D) and 48 aligned CSS contigs originating from 4B. The misjoin between 4B and 2D occurs at around 1 Mb from the scaffold start. The authors should align all the CSS contigs from the IWGSC 2014 paper and tabulate, for each scaffold, the chromosome arm assignments and genetic positions of the CSS contigs aligned to it, and determine the rate of inconsistencies. This should give a lower bound on the number of misassemblies.

Triticum 3.1 assembly is in contigs, not scaffolds, so there are no "chimeric scaffolds", although there could be chimeric joins within contigs. First we would emphasize that discrepancies found in alignments with the CSS contigs are not necessarily an indication of misassembly. They could instead could be caused by alignment artifacts due to the repetitive nature of the genome and incomplete nature of the CSS assembly: because the CSS assembly only has about 2/3 of the sequence that Triticum 3.1 has, there may be consistently aligning sequence that is absent from the CSS assembly.

We already report that our assembly has zero structural disagreements with the published *Ae tauschii* genome assembly, which represents strong validation for 4.3 Gb of the assembly (a very large proportion). However, prompted by the reviewer, we undertook an additional validation step, by aligning BAC end sequences from the TA3B BAC library to the Triticum 3.1 contigs. We describe our results in this new paragraph, in the "Assembly quality and completeness" section:

"As a further evaluation of assembly quality, we aligned 19,401 BAC ends from the wheat chromosome 3B-specific BAC library, TA3B (NCBI BioSample SAMN001187987) [14] to all contigs in Triticum 3.1. 18,465 BAC ends aligned, of which 2,739 pairs aligned to the same contig. Of these 2,739 pairs, 2,709 (99%) aligned in the correct orientation with a distance consistent with the mean size for the library. In no case did a pair of BAC ends align to a single contig in the wrong orientation. Out of all BACs where the ends aligned to different contigs, only 282 had one BAC end aligning sufficiently far from a contig's end to permit the other BAC end to align to the same contig; these could represent mis-assembled contigs, but they could also be explained by unusually long BACs or alignment artifacts."

4. The authors claim that their assembly is near-complete based on an assumed genome size of 15.34 Gb for bread wheat (Table 1). What is the authors' reference for this genome size? The often-cited paper of Arumuganathan and Earle gives the genome of Tritcum aestivum as 16 Gb. To my mind, given (i) the uncertainty in the selection of size standards and conversion factors from DNA mass into basepairs; and (ii) abundant intra-species structural variation, genome sizes should be given with an accuracy of four significant digits.

As mentioned above, we added a reference to the 16 Gb estimate of Arumuganathan and Earle. We agree that genome sizes should not be given with 4 significant digits (we assume the reviewer left out the word "not" here). We clarified that we are using 15.34 Gb "as the genome assembly size", not as the genome size.

5. The large assembly size may be due to redundant, artificially duplicated sequences. For example, there is a MEGABLAST HSP with 100 % identity and 51,645 bp alignment length between scaffolds 'scf7180004934723_0-119193' and '042698F_F_6834_008278F_F'. The authors should run a megablast search (with a large word size) of their assembly against itself to find other such potentially duplicated regions. I would not use Nucmer for this analysis because of lower sensitivity compared to megablast (at least in my hands).

Following this suggestion, we used megablast to align all assembled contigs to themselves. We then looked for all alignments of 100% identity that are longer than 50Kb. We found just two instances: the one described by the reviewer and one other, a 50,889 duplication:

```
040307F        scf7180004684127:0-64765  50889  61057   111945  64765   13877
042698F_F_6834_008278F_F      scf7180004934723:0-119193  51645  203825  255469  63706   12062
```

We have updated our assembly by removing the duplicated regions. The overall assembly size is about 100 Kb small, a tiny fraction of 15.34 Gb.

6. Were there any checks for contaminant sequence (e.g. leaf pathogens) done? Theoretically, the large assembly size can be caused by the presences of many contaminant sequences.

Yes, we ran extensive checks for contaminants, including searches against plant pathogens. When we submitted the genome to GenBank (where it is now available), NCBI also ran their own contaminant screens, which include BLAST alignments to all bacteria, viruses, vector sequences, and human. They found only a small number of contigs containing tiny bits of vector, which we trimmed accordingly. The assembly described in our manuscript represents the results after all these screens.

We would also note that we compared the k-mer content of the 3.1 assembly vs. the TGACv1 Illumina reads, and we found very few novel k-mers in the 3.1 assembly. The Illumina data used in the TGACv1 assembly is from a completely independent DNA extraction, and it was used as the source of a published assembly. This also validates that the 3.1 assembly is largely free of contaminants.

7. The hypothesis of better gene space representation (l. 304 - 305) can be easily tested: the authors should compare the representation of Chinese Spring full-length cDNAs in their assembly to previous efforts. An important quality check is also to ascertain how many of the (potentially fragmented) gene models predicted on the IWGSC 2014 assembly can be aligned to the new assembly.

As described in our response to reviewer 1, we ran BUSCO to address this question, and found that the vast majority of BUSCO genes are present in our assembly. We also compared our BUSCO results to the BUSCO results of the 2017 TGACv1 assembly.

8. In the introduction, the authors dwell on the difficulties of wheat genome sequence assembly. I would also mention the not-so-difficult aspects of wheat genomics. (i) For all practical consideration of genome assembly, wheat inbred lines do not have 6 copies of each chromosome, but three. In this regard, wheat is much easier than, for example, outcrossing tetraploid potato. (ii) Due the presence of the Pairing-of-homeologs loci (mainly Ph1), wheat behaves genetically as a diploid. (iii) The sequence divergence between the three homeologs is about 4 % in genic regions and much greater in non-genic regions. The statement regarding the

existence of "many regions of high similarity" (ll. 49-50) should be made more precise: How many regions? Which degree of similarity?

We modified the Introduction to state that the strain we sequenced "is highly inbred and thus nearly haploid, effectively reducing the number of copies of each chromosome from six to three."

9. When first reading it, I understood the sentence in ll. 293-294 as a claim that it was possible for the first to determine which sequence contigs from a wheat genome assembly originate from the D genome. This can also be done by genetic mapping with WGS data from a biparental population and has been done before (IWGSC 2014 and Chapman et al. 2015, Genome Biology). Reading the sentence for a second time, I understood that the authors only claim that theirs is the first report of subgenome assignment (in wheat) by assembly alignment, which to the best of my knowledge is true. Maybe this sentence can be rephrased for better clarity to avoid confusion.

We revised the sentence for clarity. It now reads:
"... ours is the first assembly to cleanly separate the D genome component from the A and B genomes of hexaploid wheat by aligning this assembly to the draft genome of *Aegilops tauschii*, the progenitor of the wheat D genome."

10. The authors describe the computational resource required for their assembly. I would be curious about the human resources necessary for this effort. Which skill set is required to assemble a wheat genome? What was the hands-on time? Is it possible for other research groups to conduct a similar effort without involvement of the developers of the MaSuRCA assembler?

This is a bit off-topic to include in the manuscript itself, but it should be possible for other groups that have access to the appropriate computational resources (which are very large), and that include experts in genome assembly or alignment, to replicate our results given our data, which we have made publicly available. However, for more than 20 years genome assembly has been a complex task, and scientists with little or no experience in assembly usually call upon experts to assist.

11. The author should provide more evidence that their effort has been without precedent (l. 284) (or omit this statement).

Response: we have been working in the genome assembly field for >15 years, and we have read the papers describing most of the major genomes that have been published. We have not found any reference that describes even half the amount of computing power required for this assembly (100 CPU years, as we explained in the text), and we are confident that the word "unprecedented" is accurate. If the reviewer had cited any counter-example, we would have been happy to modify this statement, but we do not believe any larger computing effort has even been dedicated to a single assembly.

12. The authors may want re-evaluate their claim about the great importance of very long reads for wheat genome assembly in light of the recently published genome assembly of wild emmer wheat (Avni et al., 2017, Science) from only Illumina data.

Actually, the just-published Avni et al paper supports our claim. Their contig N50 is 57,378 bp, while ours was over 232 Kb (4 times more contiguous). Longer reads clearly contribute a great deal to increased contiguity - a point that has been made by many other recent publications as well.

13. I concur with the editor-in-chief of Bioessays that there is no place for drama in science (see DOI:10.1002/bies.201500126), so please rephrase "dramatically" in l. 61.

We rephrased the sentence and deleted the word "dramatically". It now reads:
"By combining these very long reads with highly accurate shorter reads, we have been able to produce an assembly of the wheat genome with contigs that are more than ten times longer than those produced in any previous attempt."

14. The use of "in the end" in l. 252 can be misleading. The chromosome-based assembly published by IWGSC in 2014 was never intended as a final product, but rather as an intermediate step towards a map-based reference sequence for all chromosome arms.

We removed “in the end” from that sentence.

Reviewer #3:
The manuscript by Zimin et al. describes the whole genome sequencing of bread wheat Triticum aestivum genotype Chinese Spring and the construction of the genome assembly, using a combination of substantially long PacBio and relatively accurate high-depth Illumina reads. The manuscript thoroughly describes the assembly procedure, which is computation intensive as is the case for such genomes. I do appreciate repeated steps to polish the assembly, however, the final assembly is still quite fragmented, made up of >279K contigs. Although this assembly may represent the best near-complete bread wheat genome assembly achieved so far, I doubt that it will be of immediate use to the wheat community. One of the major shortcomings of this approach, in my opinion, is that the contigs are not readily assigned to individual chromosomes, which I believe make an assembly really useful. Although the authors identified the contigs likely belonging to the D-genome, I did not see any indication of how successful this assembly is in distinguishing homeologous sequences from the sub-genomes.

Additionally, the manuscript is mostly focused on describing the assembly procedure which is, in my opinion, quite conventional, and very few biological assessments on the genome content and organization (repeat elements, gene content etc.) are provided.

As it stands I recommend its rejection.

We agree with the reviewer that our assembly is the best near-complete bread wheat genome assembly achieved so far. However we disagree that our assembly is not of immediate use to the wheat community in its current form; indeed, many people have already asked for it based on our bioRxiv preprint. The abstract for our preprint has been accessed 3,981 times already (in just one month), and the full PDF has been downloaded 1,624 times. Its Altmetric score puts it "in the top 5% of all research outputs scored by Altmetric." Clearly there is great interest in this paper.

We would also note that the vast majority of published eukaryotic genomes do not have contigs assigned to chromosomes, although it is certainly useful to have such maps.
