# Supplementary material for: The first near-complete assembly of the hexaploid bread wheat genome, Triticum aestivum
Source: Gigascience. 2017 Oct 23;6(11):1–7. doi: 10.1093/gigascience/gix097 (PMC5691383; doi:10.1093/gigascience/gix097)
Supplement: Response-to-Reviewer-Comments_Revision-1.pdf [file gix097_response-to-reviewer-comments_revision-1.pdf]

Author's Response To Reviewer Comments

This is the final version with a few extra edits included by the Editor.
