# Supplementary material for: The first near-complete assembly of the hexaploid bread wheat genome, Triticum aestivum
Source: Gigascience. 2017 Oct 23;6(11):1–7. doi: 10.1093/gigascience/gix097 (PMC5691383; doi:10.1093/gigascience/gix097)
Supplement: Response-to-reviewers-82517.pdf [file gix097_response-to-reviewers-82517.pdf]

Dear Editors,
We have responded to all of the reviewers' comments in our revised manuscript. We thank the reviewers for their comments which helped to improve the presentation.

First we have a meta-comment. Because the reviewers are not anonymous, we discovered that all three reviewers are either current or past members of the IWGSC (Internatl Wheat Genome Sequencing Consortium). The IWGSC has a competing assembly of hexaploid wheat that they have been talking about at meetings, but that is not publicly available. They have stated that they are working on a paper, and this paper is clearly in direct competition with ours. Several of the reviewers' comments refer to this non-public genome and to their previous publications with the IWGSC. Thus as direct competitors, all 3 reviewers have a strong conflict of interest and should not have reviewed our paper; we believe they should have self-identified as being in conflict and recused themselves. However, they did not do so.

Nonetheless, we did the best we could to address all comments. Because of this strong conflict, though, and because these reviewers might be strongly motivated to delay publication of our paper, we would ask that the GigaScience editors read our responses and make a final decision without relying on the reviewers' further comments. We are very concerned that the reviewers will simply make more requests for changes and will attempt to delay our paper indefinitely, or at least until their own paper can appear.

We believe we have addressed the legitimate concerns adequately, as we explain below. We list each of the comments followed by our responses, indicating how we changed the manuscript in each case. Note that we omitted those comments not requiring a response. For clarity, the reviewers' comments are shown in italics.
Sincerely,
Steven Salzberg and Aleksey Zimin (on behalf of all the authors)
===

Reviewer #1: *In their paper, Zimin et al. present a new assembly for the wheat cultivar Chinese Spring based on a large amount of PacBio and Illumina reads. They state that this is the most complete wheat genome assembly to date (15.34 Gbp), and this makes it a potentially very valuable resource for researchers. The authors then use this assembly to align with an older Aegilops tauschii genome to separate candidate D-genome scaffolds.*
While there is no biology in this paper, it is purely technical, the advance is still of interest and the manuscript is a good fit for GigaScience as it has published quite a few technical genome assembly papers. It is very interesting that the polishing step in v3.0 removed many unique k-mers that were then reintroduced by alignment with v2.0 to generate v3.1. In their paper the authors should mention that the assembly does not include a single unknown base (N).

We thank the reviewer for these positive comments. We added a sentence to the abstract stating: "Our assembly contains no unknown (N) bases."

Some of the text is over simplistic, saying that all other attempts at assembly have failed does

not take into account of the aims of those projects which were often just to obtain the unique and low copy regions. The authors repeatedly say that this is the best assembly to date but ignore the available NRGene Chinese Spring assembly. I understand that this has not been published and so a direct comparison cannot be made, but the authors should at least acknowledge that NRGene assembly is available and compare general statistics. Saying that the wheat genome 'resisted efforts' suggests it did this deliberately. Suggesting that long reads = good, short reads = bad is again over simplistic. Repeats can be assembled with short reads when there is read pair information which is another common approach, the authors should acknowledge this and also comment on the read quality difference between long and short read sequencing. The authors claim 6 copies of each chromosome, but they should clarify that due to homozygosity, they only assemble 3 copies, A, B and D.

We rephrased the text describing the previous efforts. Instead of saying that "Multiple past attempts to assemble the genome have failed," we revised the abstract to say:
"Multiple past attempts to assemble the genome have produced assemblies that were well short of the estimated genome size."

Note that the phrase "resisted efforts to sequence it" is a common English construction, and does not mean the genome did this deliberately. This expression accurately reflects our message that the genome has been very challenging to sequence and assemble. However, we did rephrase several other statements in the introduction, as follows. To explain more clearly that the assembly contains 3 (rather than 6) copies of each chromosome, we added the following:
"All data for this assembly was generated from the Chinese spring variety (CS42, accession Dv418) of *T. aestivum*, which is highly inbred and thus nearly haploid, effectively reducing the number of copies of each chromosome from six to three."

Note that nowhere do we state that "that long reads = good, short reads = bad" as the reviewer put it. Indeed, in our previous papers we have described the benefits of various paired-end libraries, but this paper is not a review of assembly strategies, so we preferred not to engage in a lengthy digression to explain this. (One of us is a co-author of a 2010 review describing the challenges of short read sequencing – Schatz et al 2010 – which we reference in the text here.)

The reviewer mentions the "available NRGene Chinese Spring assembly" and asks for comparisons to it. The NRGene assembly is not freely or publicly available, and we do not have it. Obtaining it requires registration and signing a restrictive agreement with the IWGSC on data usage, which we will not do on principle. (Even if we did, the restrictive agreement might prevent us from including any detailed comparisons in our paper.) We also note that this genome assembly has not been published and therefore no metrics or statistics are available to which we can compare our results.

The authors use a predicted genome size of 15.3 Gbp which is on the small size for wheat genome predictions. The authors should justify the use of this number. The differences between Ae. tauschii and the bread wheat genome are suggested to be technical errors or loss or gain of

repeats. The authors should read the recent wheat pangenome paper which shows that you would expect gene presence absence variation between varieties and hence also between the diploid and polyploid.

We revised the paper to clarify (in the text and in the Table 1 caption) that we use 15.34 Gb for computing the N50 statistics for comparison of various assemblies. The computation of the N50 statistics depends critically on the assumed genome size, not the assembly size. As long as the same reasonable estimate of the genome size is used for all assemblies, contiguity comparisons are valid. We added text at the end of the introduction to make it clear that the true genome size is likely a bit larger. We now cite the flow cytometry estimate of 16Gb from (Arumuganathan and Earle, 1991). Our revised text says:
"In this paper we use 15.34 billion bases as the genome assembly size for computing the N50 statistics of different assemblies, in order to make these statistics comparable. The true genome size of bread wheat has been estimated by flow cytometry to be close to 16 Gb [5]; based on this estimate our assembly contains 96% of the genome sequence."

The main issue I have with the manuscript is the lack of quality control. The authors make statements saying that they are the first to 'reconstruct essentially all of the hexaploid wheat genome' but the assembly lacks the majority of quality control required for genome publications. Running BUSCO should be relatively straight forward and would provide a direct comparison between this and all the other assemblies (including the NRGene one and the reassembled chromosome arms from Montenegro et al. (2017)). Are all the genes identified in the previous assemblies in this one? Given that this assembly is based on long reads with low accuracy, what is the bp similarity between related portions of each of the assemblies?

We had not used BUSCO because earlier versions had failed (crashed) when we ran them on large plant genomes, but prompted by the reviewer, we downloaded the latest version (3.0.2), and it appears to work now. We then used BUSCO to compare the single copy orthologous gene content of our assembly to the TGACv1 wheat assembly, which is the best assembly published to date. The results show that our assembly completeness looks excellent, and is very slightly better than TGACv1. We added the following text to the manuscript in the Assembly quality sub-section, which we renamed to "Assembly quality and completeness":

"We used BUSCO (version 3.0.2) [14] to assess the completeness of the Triticum 3.1 assembly based on the presence of the single-copy orthologs from the OrthoDB (v9.1) [15] database. We found that 1415 out of 1440 BUSCO genes are present and complete in the Triticum 3.1 assembly, of which 161 ae single-copy and 1254 are in multiple copies. The large number of duplicated genes is likely due to the polyploidy of the genome. Only 4 BUSCO genes are fragmented and 21 are missing. We ran the same analysis on most complete published bread wheat assembly, TGACv1 [3], and found that it contains 1411 complete BUSCO genes (very slightly fewer than Triticum 3.1), of which 126 are single-copy, 1285 are multiple-copy, 8 are fragmented and 21 are missing."

Some specific issues:

Some of the language should be more precise and specific, eg. Line 61 'dramatically' better or line 62 'essentially the entire length of the genome' - this is not demonstrated. Line 89 'Most PacBio' and 'some cases', actual numbers here are important. On line 54, 'reads that contain them' should read 'reads that span them'

We revised the text, removing "dramatically better" and using precise numbers as requested. The revised text says:
"By combining these very long reads with highly accurate shorter reads, we have been able to produce an assembly of the wheat genome that is more than ten times more contigous than those produced in any previous attempt. Ours is the first assembly that contains nearly the entire length of the genome, with more than 15.3 billion bases."

It is commendable that the authors expand on the computational needs for this assembly and included some numbers for the CPU hours and walltime used as this will be very valuable data for researchers who need to justify their HPC requests. Can the researchers comment on how many nodes of the cluster the MaSuRCA process used on average and the maximum number of nodes used, if that data is available? The paper states that 'thousands of jobs' were run in parallel, is the exact number still available?

Yes, we checked the logs and found that we ran a maximum of 3320 assembly jobs at a time. In the revised text we added this detail: "thousands of jobs running in parallel (the maximum number was 3,320)"

The paper says that peak memory usage for the mega-reads assembly step was 1.2TB, however, the large memory nodes in MARCC have 1TB of memory. As far as I know MaSuRCA cannot be run in a distributed way so I do not understand where this 1.2TB memory comes from, could the researchers please comment on this?

We needed 1.2TB memory for the mega-reads computation step, which we ran on a single 1.5TB memory computer at the University of Maryland. Most readers will be unaware that MARCC's largest memory node has 1 TB (indeed we were surprised the reviewer knew this), so we didn't add this detail to the text, especially as MARCC might get larger memory nodes soon.

The methods part says that the Celera Assembler was modified to work with the authors' cluster, but the code of that modification does not seem to be available. RunCA already supports SGE clusters, was it just minor modifications on the SGE spec file, or something more complex? Minor changes wouldn't need to be shared, but if the researchers managed to (for example) get RunCA to work with SLURM (as it is used by MARCC) it would be very useful to open source these changes.

We did indeed make minor modifications to the runCA script to let it work with the SLURM scheduler. We added a sentence to the text to explain this, changing this:
"We then assembled the mega-reads and the synthetic pairs using the Celera Assembler [8] (v8.3), which was modified to work with our parallel job scheduling system."

to this:
"We then assembled the mega-reads and the synthetic pairs using the Celera Assembler [8] (v8.3), which was modified to work with our parallel job scheduling system. (The modified software is available at ftp://ftp.ccb.jhu.edu/pub/dpuiu/OTHER/SLURM/runCA.)"

Reviewer #2:

1. Although the authors assert that "full details of the experimental design and statistical methods used [were] given in the Methods section", the manuscript actually does not have a Methods section. The authors should structure their manuscript properly into Introduction, Methods, Results and Discussion sections. The Methods section should contain a description of their pipeline with full details on the software versions and parameter. A flowchart of the assembly process will improve the clarity of the manuscript.

We're not sure what the reviewer is referring to. The manuscript does not contain this phrase, nor does it contain any reference to a "Methods section." We assume this is something from the review form that the reviewer saw. Because this manuscript describes a computational result, we chose to structure it differently (as is common with computational papers) since much of the paper is about the assembly methods. In our view, restructuring the entire paper would not clarify the exposition but more likely would do just the opposite, so we left the structure as is.

2. In l. 19 of the abstract, the authors seem to have used arbitrary thresholds (i.e. assembly length > 15 Gb and N50 > 200 kb) to differentiate between success and failure of assembly efforts. Both (i) the numeric values of these cut-offs and (ii) the arbitrariness of their choice should be stated explicitly before any mention of success or failure is made. Of course, such strong judgmental terms could also simply be omitted.

We removed the statement that previous efforts failed (although one could certainly argue that they did), and instead we revised the abstract to say merely that "Multiple past attempts to assemble the genome have produced assemblies that were well short of the estimated genome size."

3. The better contiguity of the present assembly compared to previous efforts may be due to a higher rate of chimeric scaffolds, i.e. scaffolds combining sequences from physically unlinked regions. I found one such chimera: scaffold '000017F' (1.6 Mb). It has 40 aligned chromosome survey sequence (CSS) contigs originating from 2D (and POPSEQ-anchored to 2D) and 48 aligned CSS contigs originating from 4B. The misjoin between 4B and 2D occurs at around 1 Mb from the scaffold start. The authors should align all the CSS contigs from the IWGSC 2014 paper and tabulate, for each scaffold, the chromosome arm assignments and genetic positions of the CSS contigs aligned to it, and determine the rate of inconsistencies. This should give a lower bound on the number of misassemblies.

Triticum 3.1 assembly is in contigs, not scaffolds, so there are no "chimeric scaffolds", although there could be chimeric joins within contigs. First we would emphasize that discrepancies found in alignments with the CSS contigs are not necessarily an indication of misassembly. They could instead could be caused by alignment artifacts due to the repetitive nature of the genome and incomplete nature of the CSS assembly: because the CSS assembly only has about 2/3 of the sequence that Triticum 3.1 has, there may be consistently aligning sequence that is absent from the CSS assembly.

We already report that our assembly has zero structural disagreements with the published *Ae tauschii* genome assembly, which represents strong validation for 4.3 Gb of the assembly (a very large proportion). However, prompted by the reviewer, we undertook an additional validation step, by aligning BAC end sequences from the TA3B BAC library to the Triticum 3.1 contigs. We describe our results in this new paragraph, in the "Assembly quality and completeness" section:

"As a further evaluation of assembly quality, we aligned 19,401 BAC ends from the wheat chromosome 3B-specific BAC library, TA3B (NCBI BioSample SAMN001187987) [14] to all contigs in Triticum 3.1. 18,465 BAC ends aligned, of which 2,739 pairs aligned to the same contig. Of these 2,739 pairs, 2,709 (99%) aligned in the correct orientation with a distance consistent with the mean size for the library. In no case did a pair of BAC ends align to a single contig in the wrong orientation. Out of all BACs where the ends aligned to different contigs, only 282 had one BAC end aligning sufficiently far from a contig's end to permit the other BAC end to align to the same contig; these could represent mis-assembled contigs, but they could also be explained by unusually long BACs or alignment artifacts."

4. The authors claim that their assembly is near-complete based on an assumed genome size of 15.34 Gb for bread wheat (Table 1). What is the authors' reference for this genome size? The often-cited paper of Arumuganathan and Earle gives the genome of Tritcum aestivum as 16 Gb. To my mind, given (i) the uncertainty in the selection of size standards and conversion factors from DNA mass into basepairs; and (ii) abundant intra-species structural variation, genome sizes should be given with an accuracy of four significant digits.

As mentioned above, we added a reference to the 16 Gb estimate of Arumuganathan and Earle. We agree that genome sizes should not be given with 4 significant digits (we assume the reviewer left out the word "not" here). We clarified that we are using 15.34 Gb "as the genome assembly size", not as the genome size.

5. The large assembly size may be due to redundant, artificially duplicated sequences. For example, there is a MEGABLAST HSP with 100 % identity and 51,645 bp alignment length between scaffolds 'scf7180004934723_0-119193' and '042698F_F_6834_008278F_F'. The authors should run a megablast search (with a large word size) of their assembly against itself to find other such potentially duplicated regions. I would not use Nucmer for this analysis because of lower sensitivity compared to megablast (at least in my hands).

Following this suggestion, we used megablast to align all assembled contigs to themselves. We then looked for all alignments of 100% identity that are longer than 50Kb. We found just two instances: the one described by the reviewer and one other, a 50,889 duplication:

```
040307F         scf7180004684127:0-64765  50889  61057   111945  64765   13877
042698F_F_6834_008278F_F      scf7180004934723:0-119193  51645  203825  255469  63706   12062
```

We have updated our assembly by removing the duplicated regions. The overall assembly size is about 100 Kb small, a tiny fraction of 15.34 Gb.

6. Were there any checks for contaminant sequence (e.g. leaf pathogens) done? Theoretically, the large assembly size can be caused by the presences of many contaminant sequences.

Yes, we ran extensive checks for contaminants, including searches against plant pathogens. When we submitted the genome to GenBank (where it is now available), NCBI also ran their own contaminant screens, which include BLAST alignments to all bacteria, viruses, vector sequences, and human. They found only a small number of contigs containing tiny bits of vector, which we trimmed accordingly. The assembly described in our manuscript represents the results after all these screens.

We would also note that we compared the k-mer content of the 3.1 assembly vs. the TGACv1 Illumina reads, and we found very few novel k-mers in the 3.1 assembly. The Illumina data used in the TGACv1 assembly is from a completely independent DNA extraction, and it was used as the source of a published assembly. This also validates that the 3.1 assembly is largely free of contaminants.

7. The hypothesis of better gene space representation (l. 304 - 305) can be easily tested: the authors should compare the representation of Chinese Spring full-length cDNAs in their assembly to previous efforts. An important quality check is also to ascertain how many of the (potentially fragmented) gene models predicted on the IWGSC 2014 assembly can be aligned to the new assembly.

As described in our response to reviewer 1, we ran BUSCO to address this question, and found that the vast majority of BUSCO genes are present in our assembly. We also compared our BUSCO results to the BUSCO results of the 2017 TGACv1 assembly.

8. In the introduction, the authors dwell on the difficulties of wheat genome sequence assembly. I would also mention the not-so-difficult aspects of wheat genomics. (i) For all practical consideration of genome assembly, wheat inbred lines do not have 6 copies of each chromosome, but three. In this regard, wheat is much easier than, for example, outcrossing tetraploid potato. (ii) Due the presence of the Pairing-of-homeologs loci (mainly Ph1), wheat behaves genetically as a diploid. (iii) The sequence divergence between the three homeologs is about 4 % in genic regions and much greater in non-genic regions. The statement regarding the

existence of "many regions of high similarity" (ll. 49-50) should be made more precise: How many regions? Which degree of similarity?

We modified the Introduction to state that the strain we sequenced "is highly inbred and thus nearly haploid, effectively reducing the number of copies of each chromosome from six to three."

9. When first reading it, I understood the sentence in ll. 293-294 as a claim that it was possible for the first to determine which sequence contigs from a wheat genome assembly originate from the D genome. This can also be done by genetic mapping with WGS data from a biparental population and has been done before (IWGSC 2014 and Chapman et al. 2015, Genome Biology). Reading the sentence for a second time, I understood that the authors only claim that theirs is the first report of subgenome assignment (in wheat) by assembly alignment, which to the best of my knowledge is true. Maybe this sentence can be rephrased for better clarity to avoid confusion.

We revised the sentence for clarity. It now reads:
"... ours is the first assembly to cleanly separate the D genome component from the A and B genomes of hexaploid wheat by aligning this assembly to the draft genome of *Aegilops tauschii*, the progenitor of the wheat D genome."

10. The authors describe the computational resource required for their assembly. I would be curious about the human resources necessary for this effort. Which skill set is required to assemble a wheat genome? What was the hands-on time? Is it possible for other research groups to conduct a similar effort without involvement of the developers of the MaSuRCA assembler?

This is a bit off-topic to include in the manuscript itself, but it should be possible for other groups that have access to the appropriate computational resources (which are very large), and that include experts in genome assembly or alignment, to replicate our results given our data, which we have made publicly available. However, for more than 20 years genome assembly has been a complex task, and scientists with little or no experience in assembly usually call upon experts to assist.

11. The author should provide more evidence that their effort has been without precedent (l. 284) (or omit this statement).

Response: we have been working in the genome assembly field for >15 years, and we have read the papers describing most of the major genomes that have been published. We have not found any reference that describes even half the amount of computing power required for this assembly (100 CPU years, as we explained in the text), and we are confident that the word "unprecedented" is accurate. If the reviewer had cited any counter-example, we would have been happy to modify this statement, but we do not believe any larger computing effort has even been dedicated to a single assembly.

12. The authors may want re-evaluate their claim about the great importance of very long reads for wheat genome assembly in light of the recently published genome assembly of wild emmer wheat (Avni et al., 2017, Science) from only Illumina data.

Actually, the just-published Avni et al paper supports our claim. Their contig N50 is 57,378 bp, while ours was over 232 Kb (4 times more contiguous). Longer reads clearly contribute a great deal to increased contiguity - a point that has been made by many other recent publications as well.

13. I concur with the editor-in-chief of Bioessays that there is no place for drama in science (see DOI:10.1002/bies.201500126), so please rephrase "dramatically" in l. 61.

We rephrased the sentence and deleted the word "dramatically". It now reads:
"By combining these very long reads with highly accurate shorter reads, we have been able to produce an assembly of the wheat genome with contigs that are more than ten times longer than those produced in any previous attempt."

14. The use of "in the end" in l. 252 can be misleading. The chromosome-based assembly published by IWGSC in 2014 was never intended as a final product, but rather as an intermediate step towards a map-based reference sequence for all chromosome arms.

We removed "in the end" from that sentence.

Reviewer #3:
The manuscript by Zimin et al. describes the whole genome sequencing of bread wheat Triticum aestivum genotype Chinese Spring and the construction of the genome assembly, using a combination of substantially long PacBio and relatively accurate high-depth Illumina reads. The manuscript thoroughly describes the assembly procedure, which is computation intensive as is the case for such genomes. I do appreciate repeated steps to polish the assembly, however, the final assembly is still quite fragmented, made up of >279K contigs. Although this assembly may represent the best near-complete bread wheat genome assembly achieved so far, I doubt that it will be of immediate use to the wheat community. One of the major shortcomings of this approach, in my opinion, is that the contigs are not readily assigned to individual chromosomes, which I believe make an assembly really useful. Although the authors identified the contigs likely belonging to the D-genome, I did not see any indication of how successful this assembly is in distinguishing homeologous sequences from the sub-genomes.

Additionally, the manuscript is mostly focused on describing the assembly procedure which is, in my opinion, quite conventional, and very few biological assessments on the genome content and organization (repeat elements, gene content etc.) are provided.

As it stands I recommend its rejection.

We agree with the reviewer that our assembly is the best near-complete bread wheat genome assembly achieved so far. However we disagree that our assembly is not of immediate use to the wheat community in its current form; indeed, many people have already asked for it based on our bioRxiv preprint. The abstract for our preprint has been accessed 3,981 times already (in just one month), and the full PDF has been downloaded 1,624 times. Its Altmetric score puts it "in the top 5% of all research outputs scored by Altmetric." Clearly there is great interest in this paper.

We would also note that the vast majority of published eukaryotic genomes do not have contigs assigned to chromosomes, although it is certainly useful to have such maps.
