# Supplementary material for: The first near-complete assembly of the hexaploid bread wheat genome, Triticum aestivum
Source: Gigascience. 2017 Oct 23;6(11):1–7. doi: 10.1093/gigascience/gix097 (PMC5691383; doi:10.1093/gigascience/gix097)
Supplement: Reviewer-1-Report-(Original-Submission).pdf [file gix097_reviewer-1-report-(original-submission).pdf]

Reviewer Report

Title: The first near-complete assembly of the hexaploid bread wheat genome, Triticum aestivum

Version: Original Submission **Date:** 7/20/2017

Reviewer name: David Edwards

Reviewer Comments to Author:

In their paper, Zimin et al. present a new assembly for the wheat cultivar Chinese Spring based on a large amount of PacBio and Illumina reads. They state that this is the most complete wheat genome assembly to date (15.34 Gbp), and this makes it a potentially very valuable resource for researchers. The authors then use this assembly to align with an older Aegilops tauschii genome to separate candidate D-genome scaffolds.While there is no biology in this paper, it is purely technical, the advance is still of interest and the manuscript is a good fit for GigaScience as it has published quite a few technical genome assembly papers. It is very interesting that the polishing step in v3.0 removed many unique k-mers that were then reintroduced by alignment with v2.0 to generate v3.1. In their paper the authors should mention that the assembly does not include a single unknown base (N).Some of the text is over simplistic, saying that all other attempts at assembly have failed does not take into account of the aims of those projects which were often just to obtain the unique and low copy regions. The authors repeatedly say that this is the best assembly to date but ignore the available NRGene Chinese Spring assembly. I understand that this has not been published and so a direct comparison cannot be made, but the authors should at least acknowledge that NRGene assembly is available and compare general statistics. Saying that the wheat genome 'resisted efforts' suggests it did this deliberately. Suggesting that long reads = good, short reads = bad is again over simplistic. Repeats can be assembled with short reads when there is read pair information which is another common approach, the authors should acknowledge this and also comment on the read quality difference between long and short read sequencing. The authors claim 6 copies of each chromosome, but they should clarify that due to homozygosity, they only assemble 3 copies, A, B and D.The authors use a predicted genome size of 15.3 Gbp which is on the small size for wheat genome predictions. The authors should justify the use of this number. The differences between Ae. tauschii and the bread wheat genome are suggested to be technical errors or loss or gain of repeats. The authors should read the recent wheat pangenome paper which shows that you would expect gene presence absence variation between varieties and hence also between the diploid and polyploid.The main issue I have with the manuscript is the lack of quality control. The authors make statements saying that they are the first to 'reconstruct essentially all of the hexaploid wheat genome' but the assembly lacks the majority of quality control required for genome publications. Running BUSCO should be relatively straight forward and would provide a direct comparison between this and all the other assemblies (including the NRGene one and the reassembled chromosome arms from Montenegro et al. (2017)). Are all the genes identified in the previous assemblies in this one? Given that this assembly is based on long reads with low accuracy, what is the bp similarity between related portions of each of the assemblies?The authors seem to have rushed this manuscript. I understand that they may wish to publish before the expected NRGene assembly

manuscript and so have not waited for annotation or the addition of much in the way of biology, which is fine as this sort of technical manuscript is valuable, but some more precision and accuracy in the writing as well as basic quality control to justify the arguments is required.Some specific issues:Some of the language should be more precise and specific, eg. Line 61 'dramatically' better or line 62 'essentially the entire length of the genome' - this is not demonstrated. Line 89 'Most PacBio' and 'some cases', actual numbers here are important. On line 54, 'reads that contain them' should read 'reads that span them'It is commendable that the authors expand on the computational needs for this assembly and included some numbers for the CPU hours and walltime used as this will be very valuable data for researchers who need to justify their HPC requests. Can the researchers comment on how many nodes of the cluster the MaSuRCA process used on average and the maximum number of nodes used, if that data is available? The paper states that 'thousands of jobs' were run in parallel, is the exact number still available?The paper says that peak memory usage for the mega-reads assembly step was 1.2TB, however, the large memory nodes in MARCC have 1TB of memory. As far as I know MaSuRCA cannot be run in a distributed way so I do not understand where this 1.2TB memory comes from, could the researchers please comment on this?The methods part says that the Celera Assembler was modified to work with the authors' cluster, but the code of that modification does not seem to be available. RunCA already supports SGE clusters, was it just minor modifications on the SGE spec file, or something more complex? Minor changes wouldn't need to be shared, but if the researchers managed to (for example) get RunCA to work with SLURM (as it is used by MARCC) it would be very useful to open source these changes.

Level of Interest

Please indicate how interesting you found the manuscript: An exceptional article

Quality of Written English

Please indicate the quality of language in the manuscript: Acceptable

Declaration of Competing Interests

Please complete a declaration of competing interests, considering the following questions:

- Have you in the past five years received reimbursements, fees, funding, or salary from an organisation that may in any way gain or lose financially from the publication of this manuscript, either now or in the future?
- Do you hold any stocks or shares in an organisation that may in any way gain or lose financially from the publication of this manuscript, either now or in the future?
- Do you hold or are you currently applying for any patents relating to the content of the manuscript?
- Have you received reimbursements, fees, funding, or salary from an organization that holds or has applied for patents relating to the content of the manuscript?

- Do you have any other financial competing interests?
- Do you have any non-financial competing interests in relation to this paper?

If you can answer no to all of the above, write 'I declare that I have no competing interests' below. If your reply is yes to any, please give details below.

I declare that I have no competing interests

I agree to the open peer review policy of the journal. I understand that my name will be included on my report to the authors and, if the manuscript is accepted for publication, my named report including any attachments I upload will be posted on the website along with the authors' responses. I agree for my report to be made available under an Open Access Creative Commons CC-BY license (http://creativecommons.org/licenses/by/4.0/). I understand that any comments which I do not wish to be included in my named report can be included as confidential comments to the editors, which will not be published.

I agree to the open peer review policy of the journal
