# Supplementary material for: The first near-complete assembly of the hexaploid bread wheat genome, Triticum aestivum
Source: Gigascience. 2017 Oct 23;6(11):1–7. doi: 10.1093/gigascience/gix097 (PMC5691383; doi:10.1093/gigascience/gix097)
Supplement: Reviewer-2-Report-(Original-Submission).pdf [file gix097_reviewer-2-report-(original-submission).pdf]

Reviewer Report

Title: The first near-complete assembly of the hexaploid bread wheat genome, Triticum aestivum

Version: Original Submission **Date:** 7/30/2017

Reviewer name: Martin Mascher

Reviewer Comments to Author:

The manuscript of Zimin et al. describes a draft sequence assembly of hexaploid wheat from PacBio and Illumina data. The basic statistics (assembly size, N50) compare favorably to previous sequence assembly efforts in wheat. The new assembly does still not achieve the quality of a reference sequence assembly: its N50 is only 232 kb, while most wheat chromosomes are longer than 500 Mb. Moreover, neither assignment of sequence scaffolds to chromosomal locations nor gene annotation were attempted by the authors, limiting the immediate usefulness of this assembly for the wheat research community. Therefore, I do not share the authors' enthusiasm about their assembly. However, the PacBio data may become an important resource in the future to validate and improve community-based efforts to assembly a reference genome sequence for wheat. Thus, I recommend publication of this important resource in Gigascience after the authors have made the necessary major revisions described below.1. Although the authors assert that "full details of the experimental design and statistical methods used [were] given in the Methods section", the manuscript actually does not have a Methods section. The authors should structure their manuscript properly into Introduction, Methods, Results and Discussion sections. The Methods section should contain a description of their pipeline with full details on the software versions and parameter. A flowchart of the assembly process will improve the clarity of the manuscript.2. In l. 19 of the abstract, the authors seem to have used arbitrary thresholds (i.e. assembly length > 15 Gb and N50 > 200 kb) to differentiate between success and failure of assembly efforts. Both (i) the numeric values of these cut-offs and (ii) the arbitrariness of their choice should be stated explicitly before any mention of success or failure is made. Of course, such strong judgmental terms could also simply be omitted.3. The better contiguity of the present assembly compared to previous efforts may be due to a higher rate of chimeric scaffolds, i.e. scaffolds combining sequences from physically unlinked regions. I found one such chimera: scaffold '000017F' (1.6 Mb). It has 40 aligned chromosome survey sequence (CSS) contigs originating from 2D (and POPSEQ-anchored to 2D) and 48 aligned CSS contigs originating from 4B. The misjoin between 4B and 2D occurs at around 1 Mb from the scaffold start. The authors should align all the CSS contigs from the IWGSC 2014 paper and tabulate, for each scaffold, the chromosome arm assignments and genetic positions of the CSS contigs aligned to it, and determine the rate of inconsistencies. This should give a lower bound on the number of misassemblies.4. The authors claim that their assembly is near-complete based on an assumed genome size of 15.34 Gb for bread wheat (Table 1). What is the authors' reference for this genome size? The often-cited paper of Arumuganathan and Earle gives the genome of Tritcum aestivum as 16 Gb. To my mind, given (i) the uncertainty in the selection of size standards and conversion factors from DNA mass into basepairs; and (ii) abundant intra-species structural variation, genome sizes should be given with an accuracy of four significant digits.5. The large assembly size may be due to redundant, artificially

duplicated sequences. For example, there is a MEGABLAST HSP with 100 % identity and 51,645 bp alignment length between scaffolds 'scf7180004934723_0-119193' and '042698F_F_6834_008278F_F'. The authors should run a megablast search (with a large word size) of their assembly against itself to find other such potentially duplicated regions. I would not use Nucmer for this analysis because of lower sensitivity compared to megablast (at least in my hands).6. Were there any checks for contaminant sequence (e.g. leaf pathogens) done? Theoretically, the large assembly size can be caused by the presences of many contaminant sequences.7. The hypothesis of better gene space representation (l. 304 - 305) can be easily tested: the authors should compare the representation of Chinese Spring full-length cDNAs in their assembly to previous efforts. An important quality check is also to ascertain how many of the (potentially fragmented) gene models predicted on the IWGSC 2014 assembly can be aligned to the new assembly.8. In the introduction, the authors dwell on the difficulties of wheat genome sequence assembly. I would also mention the not-so-difficult aspects of wheat genomics. (i) For all practical consideration of genome assembly, wheat inbred lines do not have 6 copies of each chromosome, but three. In this regard, wheat is much easier than, for example, outcrossing tetraploid potato. (ii) Due the presence of the Pairing-of-homeologs loci (mainly Ph1), wheat behaves genetically as a diploid. (iii) The sequence divergence between the three homeologs is about 4 % in genic regions and much greater in non-genic regions. The statement regarding the existence of "many regions of high similarity" (ll. 49-50) should be made more precise: How many regions? Which degree of similarity?9. When first reading it, I understood the sentence in ll. 293-294 as a claim that it was possible for the first to determine which sequence contigs from a wheat genome assembly originate from the D genome. This can also be done by genetic mapping with WGS data from a biparental population and has been done before (IWGSC 2014 and Chapman et al. 2015, Genome Biology). Reading the sentence for a second time, I understood that the authors only claim that theirs is the first report of subgenome assignment (in wheat) by assembly alignment, which to the best of my knowledge is true. Maybe this sentence can be rephrased for better clarity to avoid confusion.10. The authors describe the computational resource required for their assembly. I would be curious about the human resources necessary for this effort. Which skill set is required to assemble a wheat genome? What was the hands-on time? Is it possible for other research groups to conduct a similar effort without involvement of the developers of the MaSuRCA assembler?11. The author should provide more evidence that their effort has been without precedent (l. 284) (or omit this statement).12. The authors may want re-evaluate their claim about the great importance of very long reads for wheat genome assembly in light of the recently published genome assembly of wild emmer wheat (Avni et al., 2017, Science) from only Illumina data.13. I concur with the editor-in-chief of Bioessays that there is no place for drama in science (see DOI:10.1002/bies.201500126), so please rephrase "dramatically" in l. 61.14. The use of "in the end" in l. 252 can be misleading. The chromosome-based assembly published by IWGSC in 2014 was never intended as a final product, but rather as an intermediate step towards a map-based reference sequence for all chromosome arms.

Level of Interest

Please indicate how interesting you found the manuscript: An article of importance in its field

Quality of Written English

Please indicate the quality of language in the manuscript: Acceptable

Declaration of Competing Interests

Please complete a declaration of competing interests, considering the following questions:

- Have you in the past five years received reimbursements, fees, funding, or salary from an organisation that may in any way gain or lose financially from the publication of this manuscript, either now or in the future?
- Do you hold any stocks or shares in an organisation that may in any way gain or lose financially from the publication of this manuscript, either now or in the future?
- Do you hold or are you currently applying for any patents relating to the content of the manuscript?
- Have you received reimbursements, fees, funding, or salary from an organization that holds or has applied for patents relating to the content of the manuscript?
- Do you have any other financial competing interests?
- Do you have any non-financial competing interests in relation to this paper?

If you can answer no to all of the above, write 'I declare that I have no competing interests' below. If your reply is yes to any, please give details below.

I have no competing interests.

I agree to the open peer review policy of the journal. I understand that my name will be included on my report to the authors and, if the manuscript is accepted for publication, my named report including any attachments I upload will be posted on the website along with the authors' responses. I agree for my report to be made available under an Open Access Creative Commons CC-BY license (http://creativecommons.org/licenses/by/4.0/). I understand that any comments which I do not wish to be included in my named report can be included as confidential comments to the editors, which will not be published.

I agree to the open peer review policy of the journal
