# Supplementary material for: The first near-complete assembly of the hexaploid bread wheat genome, Triticum aestivum
Source: Gigascience. 2017 Oct 23;6(11):1–7. doi: 10.1093/gigascience/gix097 (PMC5691383; doi:10.1093/gigascience/gix097)
Supplement: Reviewer-3-Report-(Original-Submission).pdf [file gix097_reviewer-3-report-(original-submission).pdf]

Reviewer Report

Title: The first near-complete assembly of the hexaploid bread wheat genome, Triticum aestivum

Version: Original Submission **Date:** 7/30/2017

Reviewer name: Hikmet Budak

Reviewer Comments to Author:

The manuscript by Zimin et al. describes the whole genome sequencing of bread wheat Triticum aestivum genotype Chinese Spring and the construction of the genome assembly, using a combination of substantially long PacBio and relatively accurate high-depth Illumina reads. The manuscript thoroughly describes the assembly procedure, which is computation intensive as is the case for such genomes. I do appreciate repeated steps to polish the assembly, however, the final assembly is still quite fragmented, made up of >279K contigs. Although this assembly may represent the best near-complete bread wheat genome assembly achieved so far, I doubt that it will be of immediate use to the wheat community. One of the major shortcomings of this approach, in my opinion, is that the contigs are not readily assigned to individual chromosomes, which I believe make an assembly really useful. Although the authors identified the contigs likely belonging to the D-genome, I did not see any indication of how successful this assembly is in distinguishing homeologous sequences from the sub-genomes.Additionally, the manuscript is mostly focused on describing the assembly procedure which is, in my opinion, quite conventional, and very few biological assessments on the genome content and organization (repeat elements, gene content etc.) are provided.As it stands I recommend its rejection.

Level of Interest

Please indicate how interesting you found the manuscript: An article of limited interest

Quality of Written English

Please indicate the quality of language in the manuscript: Acceptable

Declaration of Competing Interests

Please complete a declaration of competing interests, considering the following questions:

- Have you in the past five years received reimbursements, fees, funding, or salary from an organisation that may in any way gain or lose financially from the publication of this manuscript, either now or in the future?
- Do you hold any stocks or shares in an organisation that may in any way gain or lose financially from the publication of this manuscript, either now or in the future?
- Do you hold or are you currently applying for any patents relating to the content of the manuscript?

- Have you received reimbursements, fees, funding, or salary from an organization that holds or has applied for patents relating to the content of the manuscript?
- Do you have any other financial competing interests?
- Do you have any non-financial competing interests in relation to this paper?

If you can answer no to all of the above, write 'I declare that I have no competing interests' below. If your reply is yes to any, please give details below.

I declare that I have no competing interests.

I agree to the open peer review policy of the journal. I understand that my name will be included on my report to the authors and, if the manuscript is accepted for publication, my named report including any attachments I upload will be posted on the website along with the authors' responses. I agree for my report to be made available under an Open Access Creative Commons CC-BY license (http://creativecommons.org/licenses/by/4.0/). I understand that any comments which I do not wish to be included in my named report can be included as confidential comments to the editors, which will not be published.

I agree to the open peer review policy of the journal
