# Supplementary material for: The first near-complete assembly of the hexaploid bread wheat genome, Triticum aestivum
Source: Gigascience. 2017 Oct 23;6(11):1–7. doi: 10.1093/gigascience/gix097 (PMC5691383; doi:10.1093/gigascience/gix097)
Supplement: Reviewer-4-Report-(Revision-1).pdf [file gix097_reviewer-4-report-(revision-1).pdf]

Reviewer Report

Title: The first near-complete assembly of the hexaploid bread wheat genome, Triticum aestivum

Version: Revision 1 **Date:** 9/17/2017

Reviewer name: Takashi Gojobori

Reviewer Comments to Author:

I think I am a person whom you are looking for in this Editorial Board Adjudication, because I have experiences of rice genome sequencing and publication in the formation of consortium but no involvements with wheat genome sequencing.So, let me make my comments on the present case as a neutral Editorial Board member of GigaScience.I have read through Steve Salzberg's response to the three reviewers. Although his reactions to the International Wheat Genome Sequencing Consortium look a bit too strong, his response to the reviewers are quite reasonable and understandable. Indeed, it seems that he has made great attempts for revising the manuscript following the comments of the reviewers. In fact, he made revisions in many points that have been raised by the reviewers.Judging from scientific value of the present manuscript as the Data Note, I think that the present manuscript is acceptable for publication in GigaScience.

Level of Interest

Please indicate how interesting you found the manuscript: An article of importance in its field

Quality of Written English

Please indicate the quality of language in the manuscript: Acceptable

Declaration of Competing Interests

Please complete a declaration of competing interests, considering the following questions:

- Have you in the past five years received reimbursements, fees, funding, or salary from an organisation that may in any way gain or lose financially from the publication of this manuscript, either now or in the future?
- Do you hold any stocks or shares in an organisation that may in any way gain or lose financially from the publication of this manuscript, either now or in the future?
- Do you hold or are you currently applying for any patents relating to the content of the manuscript?
- Have you received reimbursements, fees, funding, or salary from an organization that holds or has applied for patents relating to the content of the manuscript?
- Do you have any other financial competing interests?
- Do you have any non-financial competing interests in relation to this paper?

If you can answer no to all of the above, write 'I declare that I have no competing interests' below. If your reply is yes to any, please give details below.

I declare that I have no competing interests

I agree to the open peer review policy of the journal. I understand that my name will be included on my report to the authors and, if the manuscript is accepted for publication, my named report including any attachments I upload will be posted on the website along with the authors' responses. I agree for my report to be made available under an Open Access Creative Commons CC-BY license (http://creativecommons.org/licenses/by/4.0/). I understand that any comments which I do not wish to be included in my named report can be included as confidential comments to the editors, which will not be published.

I agree to the open peer review policy of the journal
